# Sgs1's roles in DNA end resection, HJ dissolution, and crossover suppression require a two-step SUMO regulation dependent on Smc5/6

**DOI:** 10.1101/gad.278275.116

**Published:** 2016-06-01

**Authors:** Marcelino Bermúdez-López, María Teresa Villoria, Miguel Esteras, Adam Jarmuz, Jordi Torres-Rosell, Andres Clemente-Blanco, Luis Aragon

**Affiliations:** 1 Cell Cycle Group, MRC Clinical Sciences Centre, Imperial College, London W12 0NN, United Kingdom;; 2 Deptartment of Ciències Mèdiques Bàsiques, Institut de Recerca Biomèdica de Lleida, Universitat de Lleida, 25198 Lleida, Spain;; 3 Instituto de Biología Funcional y Genómica, Consejo Superior de Investigaciones Científicas/Universidad de Salamanca, 37007 Salamanca, Spain

**Keywords:** genome stability, homologous recombination, Sgs1–Top3, Smc complexes, Smc5/6

## Abstract

The RecQ helicase Sgs1 plays critical roles during DNA repair by homologous recombination, from end resection to Holliday junction (HJ) dissolution. Sgs1 has both pro- and anti-recombinogenic roles, and therefore its activity must be tightly regulated. However, the controls involved in recruitment and activation of Sgs1 at damaged sites are unknown. Here we show a two-step role for Smc5/6 in recruiting and activating Sgs1 through SUMOylation. First, auto-SUMOylation of Smc5/6 subunits leads to recruitment of Sgs1 as part of the STR (Sgs1–Top3–Rmi1) complex, mediated by two SUMO-interacting motifs (SIMs) on Sgs1 that specifically recognize SUMOylated Smc5/6. Second, Smc5/6-dependent SUMOylation of Sgs1 and Top3 is required for the efficient function of STR. Sgs1 mutants impaired in recognition of SUMOylated Smc5/6 (sgs1-SIMΔ) or SUMO-dead alleles (sgs1-KR) exhibit unprocessed HJs at damaged replication forks, increased crossover frequencies during double-strand break repair, and severe impairment in DNA end resection. Smc5/6 is a key regulator of Sgs1's recombination functions.

## Abstract (precis)

In this study, Bermudez-Lopez et al. investigated the molecular regulation of the RecQ helicase (Bloom/Sgs1), which plays critical roles during DNA repair by homologous recombination. The authors provide new insights into the regulation of recruitment and activation of Sgs1 at damaged sites by showing that the Sgs1 is recruited and activated at sites of DNA damage by the Smc5/6 complex through SUMOylation.

DNA is the repository of genetic information in living cells; thus, its integrity is essential for correct cellular function. The ability to repair damage to cellular DNA is therefore of paramount importance, and multiple pathways to repair DNA lesions have consequently evolved. Homologous recombination (HR) is a crucial repair pathway in mitotic cells and involves the use of similar sequences as a template for accurate repair (Paques and Haber 1999). The yeast RecQ helicase Sgs1 contributes significantly to HR (Larsen and Hickson 2013). Sgs1 forms a heteromeric complex with the type IA topoisomerase Top3 (Gangloff et al. 1994; Bennett et al. 2000; Oakley et al. 2002) and the OB-fold-containing protein Rmi1 (Chang et al. 2005; Mullen et al. 2005), a complex named STR (Sgs1–Top3–Rmi1) (Ashton and Hickson 2010). STR is involved in 5′-to-3′ resection of DNA double-strand breaks (DSBs) (Gravel et al. 2008; Mimitou and Symington 2008; Zhu et al. 2008b) and disassembles intermediates of strand invasions named D loops (van Brabant et al. 2000; Bachrati et al. 2006). In addition, STR plays a crucial role in processing late recombination intermediates during the separation of double Holliday junctions (dHJs), four-way DNA intermediates mediating covalent linkages between chromosomes that need to be resolved before chromosome segregation. In this role, Sgs1 helicase activity and Top3-unlinking ability are combined to dissolve dHJs without associated crossovers (Ira et al. 2003; Wu and Hickson 2003; Plank et al. 2006; Bzymek et al. 2010; Cejka et al. 2010). Sgs1 is also necessary to resolve recombination-dependent DNA structures that occur due to stalling and/or collapse of replication forks as they proceed through damaged DNA templates, such as that caused when cells replicate in the presence of methyl methanesulfonate (MMS) (Liberi et al. 2005). The aberrant structures are resolved by two-dimensional (2D) gel electrophoresis as cruciform X-shaped intermediates, often referred to as sister chromatid junctions (SCJ) (Liberi et al. 2005), and are close in structure to HJs (Mankouri et al. 2011).

The resolution of dHJs before the anaphase onset is an important task that somatic cells must accomplish to maintain genome integrity. In addition to STR-dependent dissolution, which is the main pathway used by cells, HJs can also be processed through nucleolytic resolution using resolvases (Sarbajna and West 2014); however, this pathway can lead to crossover or noncrossover products, depending on the orientation of the cleavage at the junction. Indeed, cells lacking Sgs1 show an elevated frequency of sister chromatid exchanges (SCEs), which is reduced by inactivation of HJ resolvases like Mus81 or Slx4 (Wechsler et al. 2011).

In meiosis, the repair machinery ensures that some recombination events initiated by programmed DSBs form crossovers between homologous chromosomes. In the absence of Sgs1 (and STR function), dramatic changes in the pattern of meiotic molecular intermediates during the repair of DSB are observed (Jessop et al. 2006; Oh et al. 2007), demonstrating a critical role for STR in meiosis.

Phenotypes similar to those of *sgs1*Δ cells have been observed upon inactivation of the SMC complex Smc5/6. These include accumulation of recombination-dependent HJs upon replication stress (Ampatzidou et al. 2006; Branzei et al. 2006; Sollier et al. 2009; Bermudez-Lopez et al. 2010) and increased crossover frequencies between sister chromatids (De Piccoli et al. 2006; Potts et al. 2006). These defects are also observed when the SUMO activity of the complex is abrogated by deletion of the C-terminal Siz/PIAS domain of Mms21, a SUMO E3 ligase subunit of Smc5/6. It is therefore likely that recombination-dependent HJs in *smc5/6* mutants are caused by defects in the SUMOylation of targets, which could either promote or repress pathways that resolve these structures.

Here we screened for substrates of the SUMO E3 ligase Mms21 in response to DNA damage. We identified subunits of the Smc5/6 and STR complexes as prominent substrates of Mms21. We show that replication stress promotes a large supercomplex between STR and Smc5/6 mediated by Sgs1 recognition of SUMOylated Smc5/6 subunits. We found that, once recruited, Sgs1 and Top3 are SUMOylated by Mms21 and demonstrate that this modification is necessary for the activity of STR during different recombination steps, crossover suppression during DSB repair, processing of SCJs at damaged replication forks, and DNA end resection of DSBs. These results demonstrate that Smc5/6 is the key regulator of the recombinogenic functions of STR.

## Results

### Multiple Smc5/6 and STR subunits are Mms21 substrates

**Figure 1.**
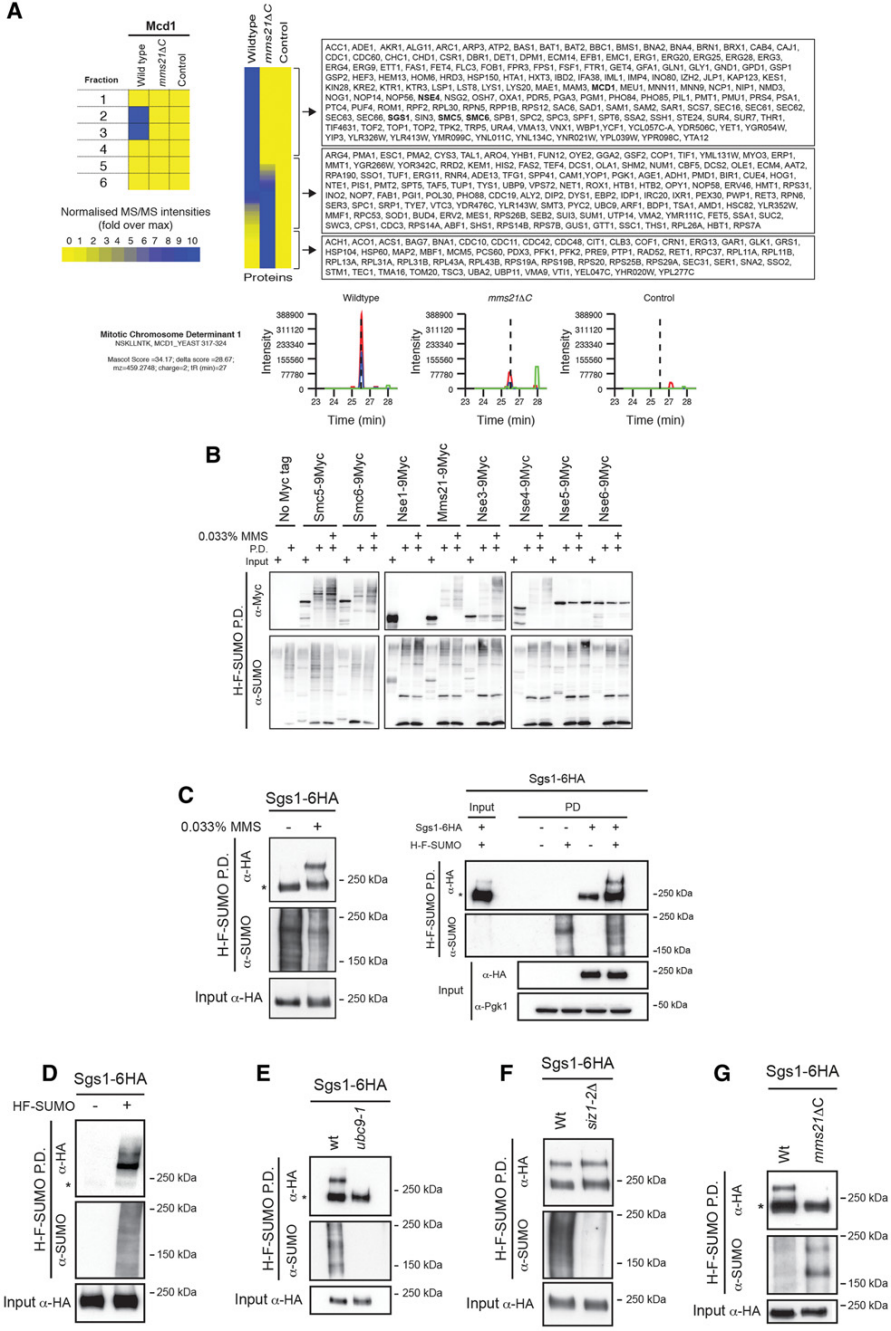
Multiple Smc5/6 subunits and Sgs1 are Mms21 substrates. (*A*) Proteomic screen to identify SUMO substrates for Mms21. Wild-type and *mms21ΔC* strains containing SUMO (SMT3) tagged with Flag and six histidines (His-Flag-SUMO [H-F-SUMO]) were exposed to 0.033% MMS. H-F-SUMO pull-downs (P.D.) from wild-type, *mms21ΔC*, and untagged cells were run on SDS gels. Lanes were separated into six fragments, and each was analyzed by nano-liquid chromatography-MS/MS (nano-LC-MS/MS). Quantitative label-free analysis allowed the identification of proteins that were specifically enriched in the pull-downs of each strain. Proteins present in pull-downs of wild-type but not *mms21ΔC* or untagged cells (the *top* list shows Mms21 substrates) represent potential Mms21 substrates. As an example, the histograms for the quantification of Mcd1 peptides in the three pull-downs are shown. Note that Mcd1 is a known target for Mms21 SUMOylation (McAleenan et al. 2012). We detected a significant enrichment of Mcd1 peptides in pull-downs from wild-type strains but not in *mms21ΔC* or untagged strains, hence validating our approach. (*B*) H-F-SUMO pull-down from wild-type cells expressing the indicated Smc5/6 subunit tagged with 9-myc. (*C*) H-F-SUMO pull-down from wild-type cells expressing Sgs1-6HA or untagged Sgs1. (*D*) Two-step H-F-SUMO pull-down from wild-type cells expressing Sgs1-6HA with or without H-F-SUMO. Proteins were purified with Ni-NTA beads, eluted, and then purified with Flag beads before loading into an SDS-PAGE gel. (*E*) H-F-SUMO pull-down from wild-type and *ubc9-1* cells expressing Sgs1-6HA. (*F*) H-F-SUMO pull-down from wild-type and *siz1-2*Δ cells expressing Sgs1-6HA. (*G*) H-F-SUMO pull-down from wild-type and *mms21ΔC* cells expressing Sgs1-6HA. In *B–G*, cells were treated with 0.033% MMS for 2 h before colleting them. An asterisk indicates the unmodified form of Sgs1.

We sought to identify targets of the SUMO E3 ligase Mms21 in response to DNA damage. To this aim, we used a proteomics approach. We tagged Smt3 (*Saccharomyces cerevisiae* homolog of SUMO) with the 6his-Flag tag in wild-type and *mms21ΔC* cells, a mutant that lacks the C-terminal Siz/PIAS domain of Mms21, and performed pull-down experiments in cells treated with 0.033% MMS for 2 h. Proteins eluted from the columns were run on SDS-PAGE gels, and individual lanes were excised into six fragments (Fig. 1A), digested with trypsin, and analyzed by tandem mass spectrometry (MS/MS). We identified >150 SUMOylated proteins on MMS-treated wild-type cells that were absent in *mms21ΔC* or control samples (Fig. 1A). Among the potential Mms21 substrates were proteins previously characterized as bona fide targets, such as subunits of the Smc5/6 complex (Andrews et al. 2005; Zhao and Blobel 2005) and the kleisin of cohesin, Mcd1/Scc1 (Fig. 1A; McAleenan et al. 2012).

The Smc5/6 subunits Smc5, Smc6, and Nse4 were in the list of proteins enriched in the pull-downs of wild-type cells over *mms21ΔC* and controls (Fig. 1A). First, we decided to confirm that these Smc5/6 subunits were SUMOylated by Mms21. To this aim, we tagged Smt3 with the 6his-Flag tag and individual Smc5/6 subunits with the 9-myc tag in wild-type and *mms21ΔC* backgrounds. As previously reported, Smt3 pull-downs revealed that the SUMOylation of the Smc5/6 complex subunits Smc5, Smc6, and Nse4 are Mms21-dependent (Supplemental Fig. S1A–C). We then proceeded to investigate what subunits of the Smc5/6 complex are SUMOylated in response to MMS. We generated strains where, in addition to Smt3 (6his-Flag-SMT3), individual Smc5/6 subunits were tagged and performed Smt3 pull-downs in the presence and absence of 0.033% MMS. We found that, in addition to Smc5, Smc6, and Nse4, Nse2 and Nse3 are also SUMOylated (Fig. 1B). These subunits were mildly modified in the absence of DNA damage, but their SUMOylation was enhanced upon MMS treatment (Fig. 1B).

Among the proteins identified in the MMS-treated SUMO pull-downs of wild-type cells that were absent in *mms21ΔC* and control samples was the RecQ helicase Sgs1 (Fig. 1A). We decided to test whether the identification of Sgs1 was correct. We tagged Smt3 and Sgs1 and performed Smt3 pull-downs as before. We detected high-molecular-weight forms of Sgs1 in our SUMO pull-downs in the presence of MMS (Fig. 1C). These were dependent on the Smt3 tag (Supplemental Fig. S2); however, nonspecific binding of Sgs1 to the beads was observed (Fig. 1C; Supplemental Fig. S2), which prompted us to use a two-step Smt3 pull-down with the histidine and Flag tags. Using this approach, we could prevent pull-down of unmodified Sgs1, confirming that the high-molecular-weight band observed is indeed SUMO forms of Sgs1 (Fig. 1D). In addition, inactivation of the E2 SUMO-conjugating activity of Ubc9 prevented the high-molecular-weight band in single pull-downs (Fig. 1E), confirming that this band represents SUMOylation. These results are consistent with previous studies (Wohlschlegel et al. 2004; Branzei et al. 2006; Lu et al. 2010) and show that Sgs1 is SUMOylated in the presence of DNA damage.

**Figure 2.**
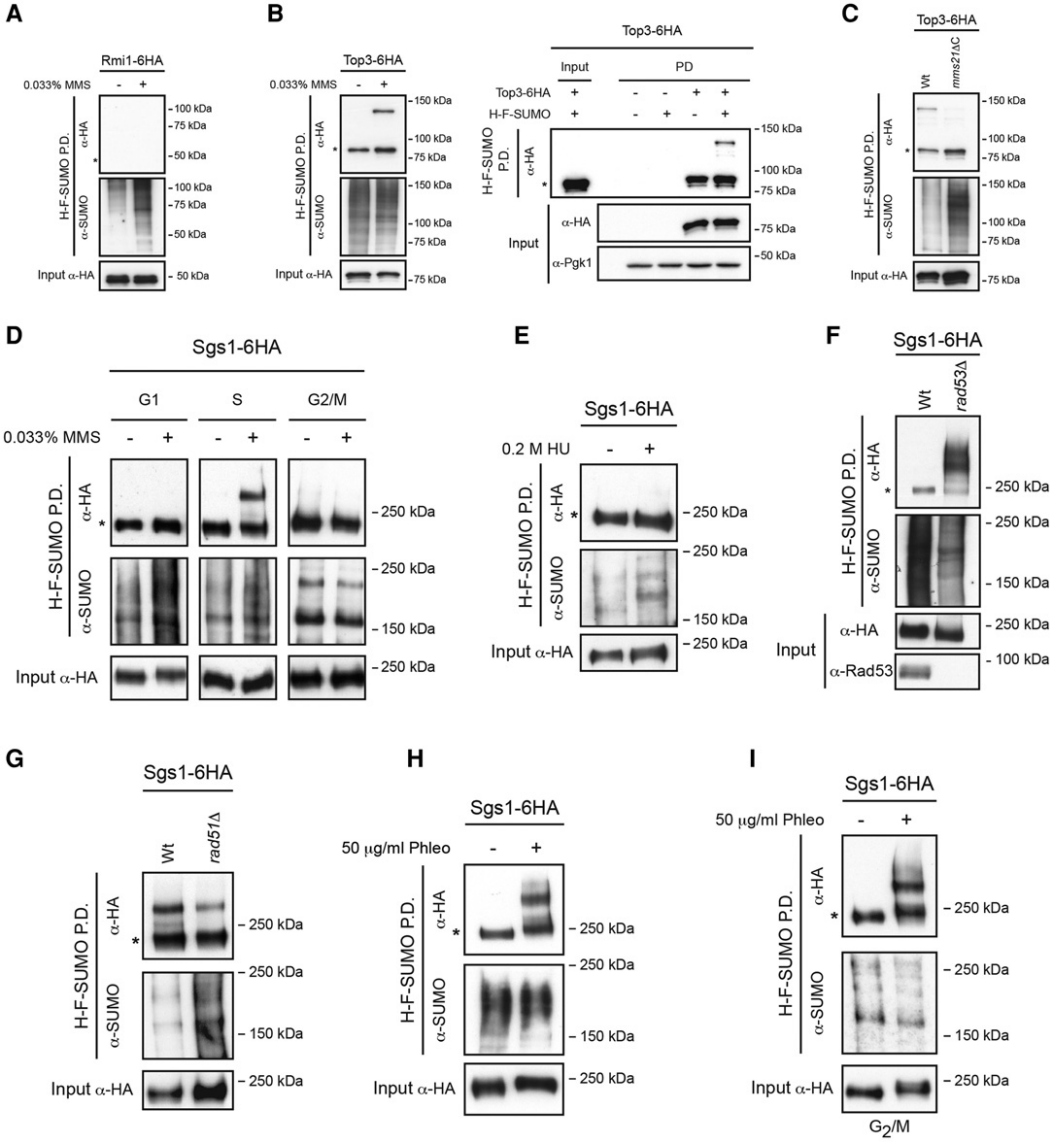
Recombinational repair triggers Sgs1 SUMOylation. (*A*) His-Flag-SUMO (H-F-SUMO) pull-down (P.D.) from wild-type cells expressing Rmi1-6HA. (*B*) H-F-SUMO pull-down from wild-type cells expressing Top3-6HA or untagged Top3. (*C*) H-F-SUMO pull-down from wild-type and *mms21ΔC* cells expressing Top3-6HA. (*D*) H-F-SUMO pull-down from wild-type cells expressing Sgs1-6HA. Cells were arrested in G1 with α factor and in G2/M with nocodazole. Once arrested, cell cultures were split into two, and one half was treated with 0.033% MMS for 2 h before collection. All cultures were maintained arrested. Cells treated in S phase were released from G1 arrest, allowing them to enter into S phase with or without 0.033% MMS. (*E*) H-F-SUMO pull-down from wild -type cells expressing Sgs1-6HA. (*F*) H-F-SUMO pull-down from wild-type and *rad53*Δ cells expressing Sgs1-6HA. Cells were treated with 0.2 M hydroxyurea (HU) for 2 h before collection. (*G*) H-F-SUMO pull-down from wild-type and *rad51*Δ cells expressing Sgs1-6HA. (*H*) H-F-SUMO pull-down from wild-type cells expressing Sgs1-6HA. (*I*) H-F-SUMO pull-down from wild-type cells expressing Sgs1-6HA. Cells were arrested in G2/M with nocodazole. Once arrested, cells were treated with phleomycin (phleo) for 2 h and maintained under nocodazole arrest. Where indicated, cells were treated with 0.033% MMS (*A*–*D*,*G*), 0.2 M HU (*E*,*F*), or 50 μg/mL phleomycin (*H*,*I*) for 2 h before collection. An asterisk indicates the unmodified form of the proteins.

Next, we sought to investigate which of the E3 ligases in yeast is responsible for Sgs1 SUMOylation. We observed normal levels of Sgs1 SUMOylation in cells lacking the Siz1 and Siz2 ligases (Fig. 1F), while no Sgs1 SUMO forms were detected in *mms21ΔC* cells (Fig. 1G). Previous studies had shown that Sgs1 SUMOylation is independent of Mms21 function (Branzei et al. 2006); however, these studies had used higher dosage of MMS (0.3% rather than 0.033%). We next tested whether Sgs1 is also SUMOylated in an Mms21-dependent manner when cells are exposed to 0.3% MMS (Supplemental Fig. S3A,B). We found that at such high doses, the bulk of Sgs1 SUMOylation depends on Mms21; however, we detected residual Sgs1 SUMOylation in *mms21ΔC* cells (Supplemental Fig. S3B), explaining the discrepancy. Our results therefore confirm the proteomic screen (Fig. 1A) in which Sgs1 was identified as a target for Mms21 SUMOylation. Next, we sought to investigate whether other subunits of the STR complex are SUMOylated. We generated strains carrying tags in Smt3 and Top3 or Rmi1 and performed SUMO pull-downs. Rmi1 SUMO forms were not detected (Fig. 2A), however, Top3 SUMO forms were observed in the presence of MMS (Fig. 2B). Importantly, Top3 SUMOylation was absent in *mms21ΔC* cells (Fig. 2C). We conclude that both the Smc5/6 and STR complexes are extensively SUMOylated by Mms21 in response to DNA damage.

### Recombinational repair triggers Sgs1 SUMOylation

Sgs1 SUMOylation was observed in the presence of DNA alkylation damage caused by exposure to MMS (Fig. 1C). This agent causes damage at replication forks (Tercero et al. 2003), requiring active repair to proceed through replication (Tercero and Diffley 2001). Indeed, we observed Sgs1 SUMO forms only in cells treated with MMS during S phase (Fig. 2D). Hydroxyurea (HU) is an inhibitor of the ribonucleotide reductase that halts replication forks by limiting dNTP pools, but restart of these paused forks occurs normally when HU is removed from the medium (Sogo et al. 2002). Surprisingly, HU treatment did not cause Sgs1 SUMOylation (Fig. 2E). One of the major differences between the cellular response to MMS and HU damage in S phase relies on the fact that MMS triggers HR repair (Xiao et al. 1996), while, in response to HU, HR is inhibited by the S-phase checkpoint kinases Mec1 and Rad53 (Sogo et al. 2002). We therefore considered the possibility that Sgs1 SUMOylation is linked to HR. To explore this, we tested Sgs1 SUMOylation in cells lacking the checkpoint kinase Rad53 arrested with HU, which leads to collapse of HU-stalled forks triggering recombinational repair. Consistent with our hypothesis, we observed a dramatic up-regulation of Sgs1 SUMOylation in *rad53*Δ cells exposed to HU (Fig. 2F). Next, we reasoned that if HR triggers Sgs1 SUMOylation, then impairing this repair pathway should decrease Sgs1 SUMOylation levels. Indeed, we found supporting evidence for this, as Sgs1 SUMOylation levels were reduced in *rad51*Δ cells exposed to DNA damage (Fig. 2G; Supplemental Fig. S4A). Treating cells with the DSB-inducing drug phleomycin, which triggers HR-dependent repair pathways, led to Sgs1 SUMOylation (Fig. 2H) even in cells arrested in G2/M (Fig. 2I). Moreover, we used a galactose-inducible system to deliver a single irreparable DSB at the MAT locus on chromosome III (Sugawara and Haber 2006) and tested the effect on Sgs1 SUMOylation (Supplemental Fig. S4B). Interestingly, Sgs1 was SUMOylated in response to a single DSB in G2-arrested cells and, to a lesser extent, G1-blocked cells (Supplemental Fig. S4B). These results demonstrate that Sgs1 SUMOylation occurs when cells commit to recombinational pathways (including DSB resection) for repair of DNA damage.

### Smc5/6 and STR form a large complex under DNA damage conditions

**Figure 3.**
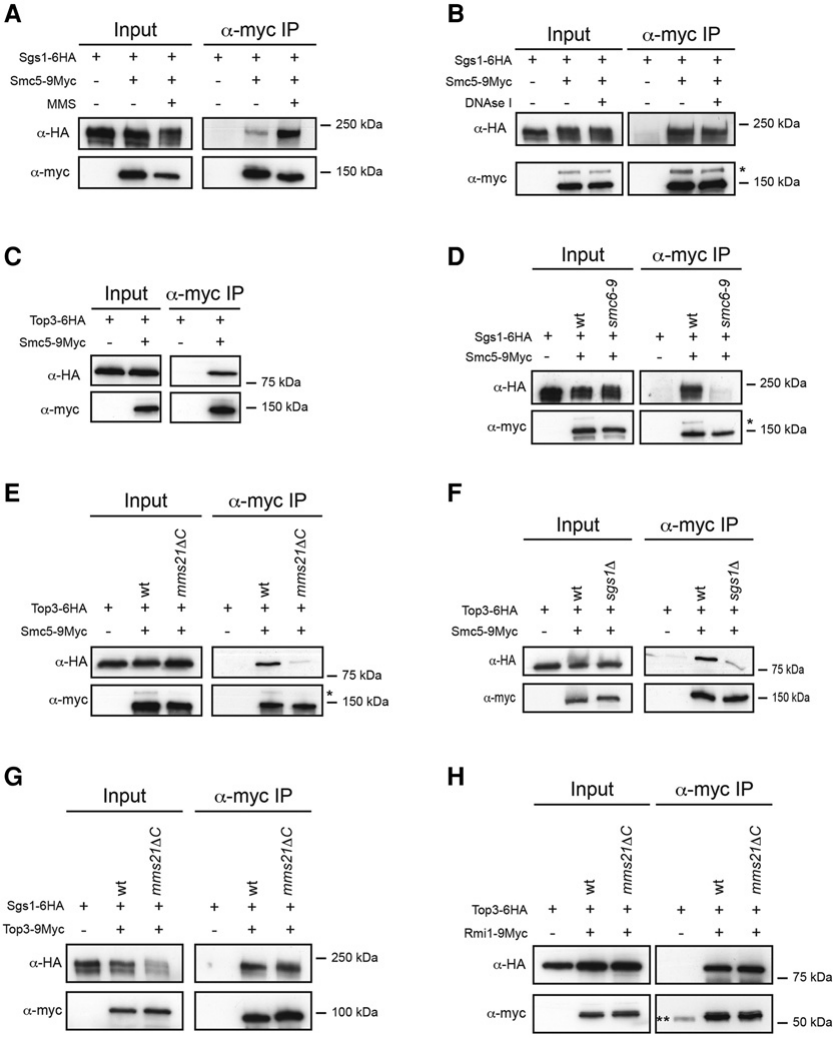
Sm5/6 and STR form a large complex under DNA damage conditions. (*A*) Analysis of the Smc5–Sgs1 interaction. (*B*) Analysis of the Smc5–Sgs1 interaction. Protein extracts were treated with DNase I before Smc5-9myc was immunoprecipitated. (*C*) Analysis of the Smc5–Top3 interaction. (*D*) Analysis of the Smc5–Sgs1 interaction in wild-type and *smc6-9* cells. Cells were shifted for 30 min to 37°C to inactivate *smc6-9* allele before addition of MMS. After 2 h, cells were collected, and the Smc5–Sgs1 interaction was determined. (*E*) Analysis of the Smc5–Top3 interaction in wild-type and *mms21ΔC* cells. (*F*) Analysis of the Smc5–Top3 interaction in wild-type and *sgs1*Δ cells. (*G*) Analysis of the Sgs1–Top3 interaction in wild-type and *mms21ΔC* cells. (*H*) Analysis of the Rmi1–Top3 interaction in wild-type and *mms21ΔC* cells. In *A*–*H*, cells were treated with 0.033% MMS for 2 h before collection. Smc5-9myc (*A*–*F*), Top3-9myc (*G*), or Rmi1-9myc (*H*) was immunoprecipitated, and the coimmunoprecipitation of Sgs1-6HA (*A*,*B*,*D*,*G*) or Top3-6HA (*C*,*E*,*F*,*H*) was analyzed by Western blot. An asterisk indicates SUMOylated Smc5-9myc. The double asterisk indicates an unspecific band detected during the immunoprecipitation.

The SUMOylation of the STR subunits Sgs1 and Top3 by Mms21 in response to DNA damage (Figs. 1G, 2C) prompted us to investigate a possible interaction between the STR and Smc5/6 complexes. First, we generated yeast strains expressing tagged copies of Smc5 (Smc5-9myc) and Sgs1 (Sgs1-6HA). Pull-downs of Smc5 resulted in coimmunoprecipitation of Sgs1-6HA when cells were exposed to MMS (Fig. 3A). Treatment with DNase I did not prevent coimmunoprecipitation of Sgs1 (Fig. 3B), demonstrating that DNA does not mediate the interaction. Top3 also coimmunoprecipitated with Smc5 (Fig. 3C), confirming the interaction of the STR complex as opposed to Sgs1 individually. We then investigated whether Smc5/6 integrity is required for the interaction of these two complexes; we used the conditional *smc6-9* allele for this purpose. Inactivation of Smc6 prevented Smc5 interaction with Sgs1 (Fig. 3D). Interestingly, this correlates with a drastic reduction in the SUMOylation levels of Sgs1 after Smc6 inactivation (Supplemental Fig. S5). Moreover, abrogating the SUMO E3 activity of Smc5/6 through *mms21ΔC* also prevented the interaction (Fig. 3E), demonstrating that SUMOylation is required for the interaction of the two complexes. In addition, we found that Smc5 and Top3 no longer coimmunoprecipitate in *sgs1*Δ cells (Fig. 3F). Importantly, Smc5/6 is not involved in mediating interaction between STR subunits, as the complex was intact in cells compromised in Smc5/6 function (Fig. 3G,H; Supplemental Fig. S6A,B). We conclude that the Smc5/6 and STR complexes form a large complex in the presence of DNA damage and that intact individual complexes and Mms21-dependent SUMOylation are required for this.

### SUMO-interacting motifs (SIMs) in Sgs1 mediate interaction with Smc5/6

**Figure 4.**
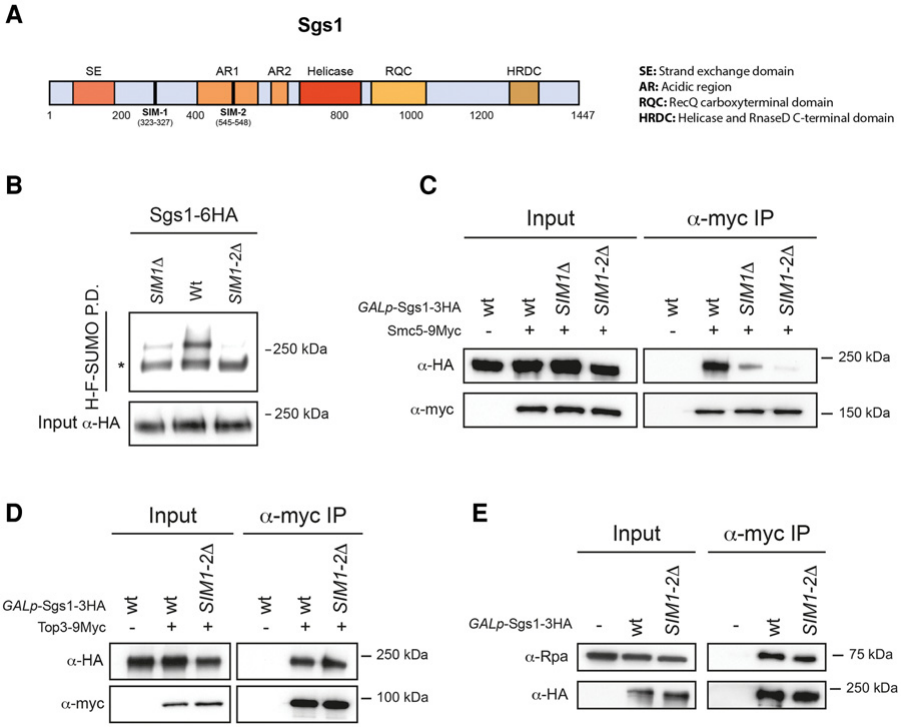
Sgs1 SIMs mediate Smc5/6 and STR interaction. (*A*) Schematic representation of the functional motifs and domains of Sgs1. (*B*) His-Flag-SUMO (H-F-SUMO) pull-down (P.D.) from cells expressing wild-type, *SIM1*Δ, and *SIM1-2*Δ Sgs1-6HA. (*C*) Analysis of the Smc5–Sgs1 interaction. (*D*) Analysis of the Sgs1–Top3 interaction. (*E*) Analysis of the Sgs1–Rpa interaction. In *B*–*E*, cells were treated with 0.033% MMS for 2 h before collection. In *C*–*E*, cells were grown in YP raffinose overnight. Next, galactose was added to a final concentration of 2% to induce Sgs1-3HA expression for 2 h before collection. Smc5-9myc (*C*), Top3-9myc (*D*), or Sgs1-3HA (*E*) was immunoprecipitated, and the coimmunoprecipitation of Sgs1-3HA (*C*,*D*) or Rpa1 (*E*) was analyzed by Western Blot. An asterisk indicates the unmodified form of Sgs1.

The mammalian homolog of Sgs1, BLM, is also a SUMO substrate (Eladad et al. 2005). Interestingly, the SUMOylation requires two SIMs in BLM (Zhu et al. 2008a). Analogously to BLM, we found two SIMs within Sgs1, at amino acid positions 323–327 and 545–548 (Fig. 4A). This prompted us to investigate whether, like BLM (Zhu et al. 2008a), Sgs1's SIMs contribute to its SUMOylation. We mutated the SIM located within amino acids 323–327 (*sgs1-SIM1*Δ) alone or in combination with the SIM spanning amino acids 545–548 (*sgs1-SIM1-2*Δ) to alanines to disrupt the noncovalent interaction with SUMOylated proteins and tested the ability of these mutants to be SUMOylated in response to DNA damage. We found a significant reduction in SUMOylation in *sgs1-SIM1*Δ (Fig. 4B) and a full block in *sgs1-SIM1-2*Δ compared with wild-type Sgs1 (Fig. 4B). Therefore, like BLM, Sgs1's SIMs are necessary for its SUMO modification. Since Smc5/6 is itself SUMOylated (Fig. 1B) and interacts with STR in response to DNA damage (Fig. 3A,B), we wondered whether Sgs1's SIMs serve to recognize SUMOylated Smc5/6 to mediate the interaction between the STR and Smc5/6 complexes. To investigate this possibility, we tested whether deleting Sgs1 SIMs affected the interaction with Smc5/6. We expressed *sgs1-SIM1*Δ and *sgs1-SIM1-2*Δ mutants in cells experiencing DNA damage where Smc5 was also tagged. Coimmunoprecipitation of wild-type Sgs1 with Smc5 was observed as before (Fig. 4C); however, the interaction weakened in *sgs1-SIM1*Δ (Fig. 4C) and was abolished in *sgs1-SIM1-2*Δ (Fig. 4C). Importantly, Sgs1 SIMs are not required for Sgs1's interaction with Top3 (Fig. 4D) or its interaction with other SUMOylated proteins, like RPA (Fig. 4E). From these results, we conclude that Sgs1's SIMs are specifically involved in the recognition of SUMOylated Smc5/6 and necessary for the binding of STR to Smc5/6.

### Sgs1 SUMOylation occurs at multiple lysine residues

**Figure 5.**
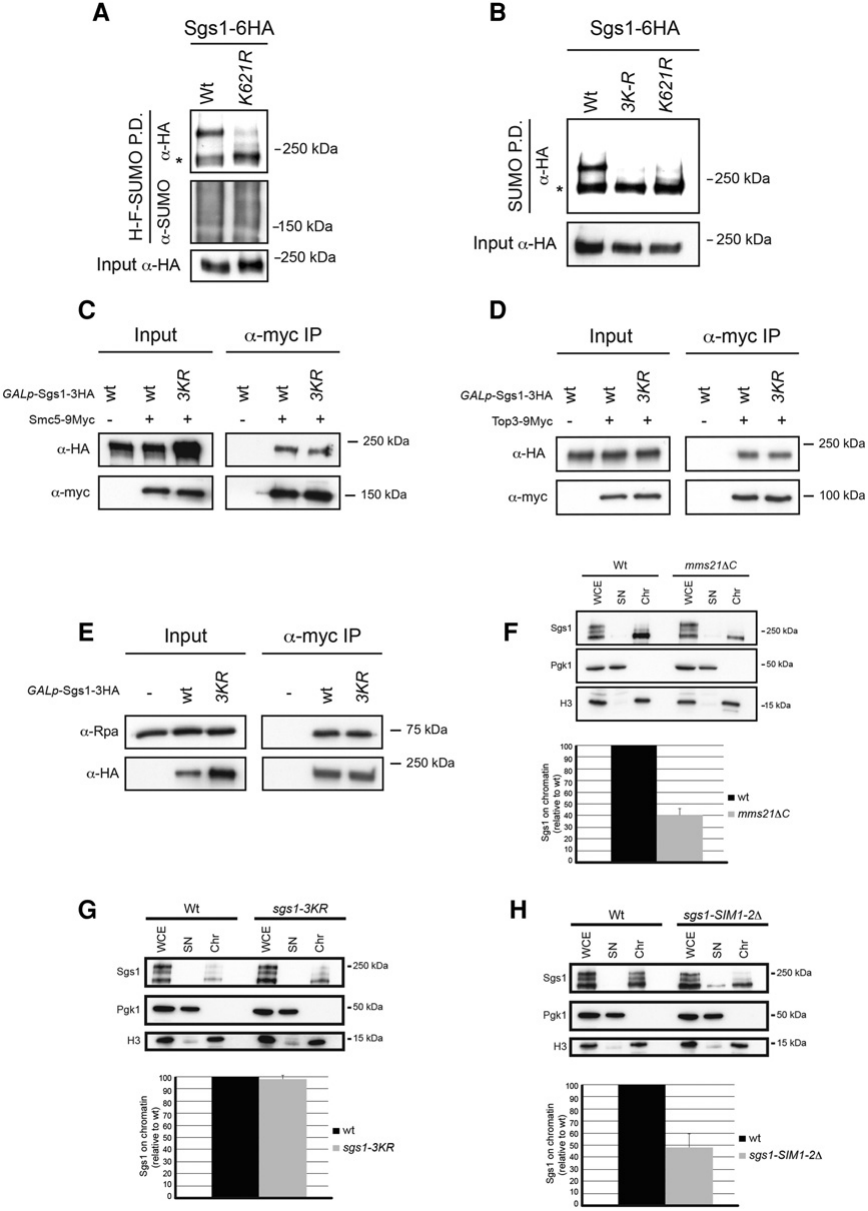
Sgs1 SUMOylation occurs at multiple lysine residues, and the Sgs1 interaction with Smc5/6 is required for its recruitment to chromatin. (*A*) His-Flag-SUMO (H-F-SUMO) pull-down (P.D.) from cells expressing wild-type and *sgs1-K621R-6HA*. (*B*) H-F-SUMO pull-down from cells expressing wild-type, *K621R*, and *K175/621/831R* (*3KR*) Sgs1-6HA. (*C*) Analysis of the Sgs1–Smc5 interaction. (*D*) Analysis of the Sgs1–Top3 interaction. (*E*) Analysis of the Sgs1–Rpa1 interaction. In *A*–*H*, cells were treated with 0.033% MMS for 2 h before collection. Smc5-9myc (*C*), Top3-9myc (*D*), or Sgs1-3HA (*E*) was immunoprecipitated, and the coimmunoprecipitation of Sgs1-3HA (*C*,*D*) or Rpa1 (*E*) was analyzed by Western blot. (*F*) Chromatin fractionation assay from wild-type and *mms21*ΔC cells expressing Sgs1-6HA. (*G*) Chromatin fractionation assay from wild-type cells expressing Sgs1-3HA wild type or *sgs1-3KR*. (*H*) Chromatin fractionation assay from wild-type cells expressing Sgs1-3HA wild type or *sgs1-SIM1-2*Δ. In *G* and *H*, cells were grown in YP raffinose overnight. Next, galactose was added to a final concentration of 2% to induce Sgs1-3HA expression for 2 h before collection. In *A*–*H*, cells were treated with 0.033% MMS for 2 h before collection. In *F*–*H*, controls for a chromatin-bound protein (histone H3) and cytoplasmic soluble (3-phosphoglycerate kinase; Pgk1) are shown. Quantifications of the chromatin fractionation assays are shown. The abundance of Sgs1 on chromatin was normalized with histone H3 as an internal loading control in our blots. Mean values and standard deviations of two independent experiments are shown. Nonsaturated exposures were used for gel quantifications using ImageJ. (WCE) Whole-cell extract; (SN) supernatant; (Chr) chromatin fraction.

The Sgs1 protein sequence contains three lysine residues that lie within SUMO consensus sites (ΨKxE) at amino acid positions 175, 621, and 831. Lys621 has been reported to be the conjugation site (Lu et al. 2010); however, SUMO-defective Sgs1 (*sgs1-K621R*) mutants were reported to be impaired only in recombination between telomeres (Lu et al. 2010). In contrast, BLM is SUMOylated at two lysine residues, K317 and 331, and cells expressing the SUMO-defective BLM (BLM-SD) display high levels of SCEs (Eladad et al. 2005). During our pull-down analyses, a major SUMO form was observed (Fig. 1D); however, higher-molecular-weight bands were also visible in some experiments (Fig. 1D; Supplemental Fig. S2). This was also the case when Sgs1 SUMOylation was tested in *rad53*Δ cells exposed to HU (Fig. 2F). Therefore, Sgs1 is likely to be modified in lysines other than K621. To evaluate this, we generated Sgs1 alleles where the main SUMO reported site at Lys621 was mutated to arginine (*sgs1-K621R*) and investigated the SUMOylation of this mutant protein. Consistent with previous reports, we observed a dramatic reduction in SUMOylation for *sgs1-K621R* compared with wild-type Sgs1 (Fig. 5A), confirming that Lys621 in Sgs1 is indeed an important SUMO acceptor site. However, SUMO forms were still detectable in the *sgs1-K621R* pull-downs (Fig. 5A). We then mutated the three lysines in Sgs1 that lie within SUMOylation consensus sites to arginines (*sgs1-K175/621/831R*). We observed a further reduction in SUMOylation levels in this triple mutant (which we refer to as *sgs1-3KR*) compared with *sgs1-K621R* (Fig. 5B). This result demonstrates that, although Lys621 in Sgs1 is the main acceptor site for SUMO, as reported earlier (Lu et al. 2010), modification also occurs at Lys175 and Lys831.

Next, we sought to investigate whether Sgs1 SUMOylation is necessary for the physical interaction between the STR and Smc5/6 complexes. We tested the ability of the SUMO-deficient *sgs1-3KR* protein to interact with Smc5 and found no defects (Fig. 5C). We also observed no defects in the interaction with Top3 (Fig. 5D) and RPA (Fig. 5E). These results suggest that Sgs1 (and Top3) SUMOylation by Mms21 occurs following the Sgs1 SIM-mediated interaction of STR and Smc5/6.

### Sgs1 interaction with the Smc5/6 complex is required for its recruitment to chromatin

SUMOylation of BLM is important for its correct nuclear localization and dynamics (Eladad et al. 2005). We therefore tested whether Sgs1 SUMOylation is also important for its recruitment to chromatin. To this aim, we first investigated what fraction of Sgs1 is bound to chromatin in the presence of DNA damage using chromatin-binding assays. Cells expressing wild-type Sgs1 were exposed to MMS, and the presence of the helicase was probed in soluble and chromatin fractions of the extracts. We found that all detectable Sgs1 was present in the chromatin-bound fraction (Fig. 5F). Histone H3 and 3-phosphoglycerate kinase (Pgk1) were present in the chromatin-bound or soluble fraction, respectively (as well as being detected in whole-cell extracts), thus validating our fractionation assay. Next, we performed chromatin-binding assays of Sgs1 in the *mms21ΔC* genetic background. Interestingly, a dramatic reduction in chromatin-bound Sgs1 was observed (Fig. 5F), and, unlike in wild-type cells, Sgs1 was also detected in the soluble fraction in the *mms21ΔC* samples (Fig. 5F). Importantly, Sgs1 SUMOylation is not necessary for its recruitment to chromatin, since *sgs1-3KR* was recruited to chromatin at similar levels, compared with Sgs1 (Fig. 5G; Supplemental Fig. S7). On the other hand, disrupting the interaction between STR and Smc5/6 in the *sgs1-SIM1-2*Δ reduced Sgs1 chromatin binding significantly (Fig. 5H). Chromatin binding of Smc5 was not affected in the *mms21ΔC* or *sgs1*Δ genetic backgrounds (Supplemental Fig. S8A,B), demonstrating that Smc5/6 recruitment to chromatin is independent of its SUMOylation state or its interaction with the STR complex. From these results, we conclude that Sgs1 interaction with SUMOylated Smc5/6 is important for the recruitment of the helicase to chromatin in the presence of DNA damage.

### Sgs1 SUMOylation prevents accumulation of joint molecules at damaged replication forks

**Figure 6.**
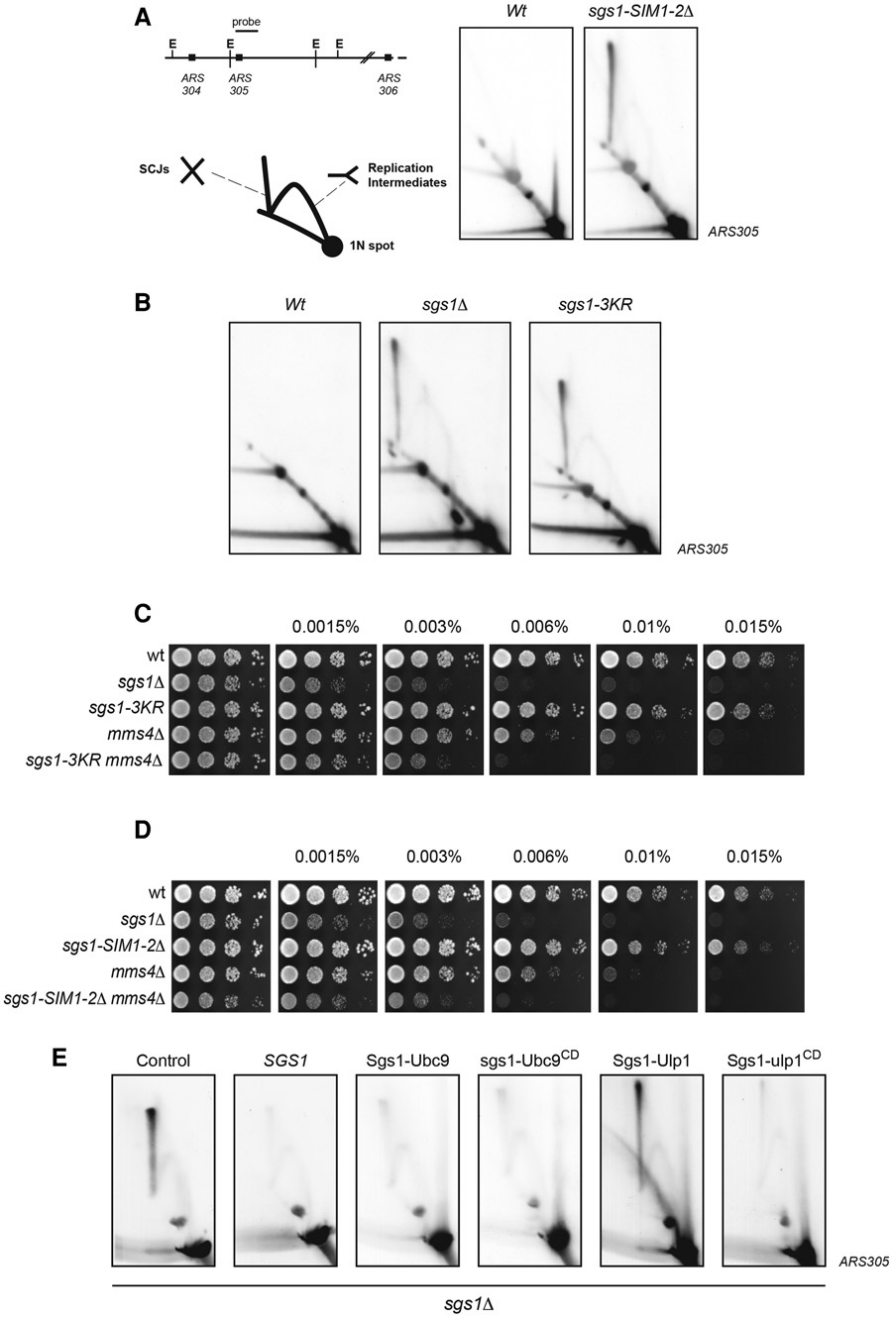
Sgs1 SUMOylation promotes dissolution of HJs at damaged replication forks. (*A*, *left* panel) Genomic region containing *ARS305* origin of replication and the probe used for hybridization. Schematic representation of structures visualized by 2D gel electrophoresis. (*Right* panel) 2D gel electrophoresis of wild-type and *sgs1-SIM1-2*Δ cells. Cells were arrested in G1 with α factor. Once arrested, cells were released from G1 arrest into fresh medium containing 0.033% MMS for 3 h before samples were taken and processed for 2D gel. Note the accumulation of SCJs in the mutant strain. (*B*) 2D gel electrophoresis of wild-type, *sgs1*Δ, and *sgs1-3KR* cells. Cells were treated as in *A*. Note that sgs1-3KR accumulates SCJs similar to *sgs1*Δ cells. (*C*) Growth test analysis of wild-type and *sgs1*Δ, *sgs1-3KR*, *mms4*Δ, and *sgs1-3KR mms4*Δ mutant cells. Plates were incubated at 30°C in the presence of the indicated MMS concentration. (*D*) Growth test analysis of wild-type and *sgs1*Δ, *sgs1-SIM1-2*Δ, *mms4*Δ, and *sgs1-SIM1-2Δ mms4*Δ mutant cells. Plates were incubated at 30°C in the presence of the indicated MMS concentration. (*E*) 2D gel electrophoresis of *sgs1*Δ cells expressing the indicated Sgs1 fusion construct. Cells were grown in YP raffinose overnight. Cells were arrested in G1 with α factor. Once arrested, cells were released into fresh medium containing 0.033% MMS and galactose at 2% final concentration to induce the expression of the constructs for 3 h before being processed for 2D gel. Note the presence of SCJs in the control and the Sgs1-Ulp1 fusion. (CD) Catalytically dead.

Sgs1 is important for the processing of recombination-dependent structures at damaged replication forks (Liberi et al. 2005). These intermediates require Rad51 and are observed when *sgs1*Δ cells replicate in the presence of MMS (Liberi et al. 2005). Using 2D gel electrophoresis, a spike signal for these X-shaped SCJs or HJs can be observed (Liberi et al. 2005; Mankouri et al. 2011). Our analysis revealed that Sgs1 SUMOylation, like the replication-dependent joint molecules (Liberi et al. 2005), is affected by the presence of Rad51 in cells (Fig. 2G). This prompted us to investigate whether Sgs1 interaction with Smc5/6 contributes to its role in processing HJs at damaged replication forks by probing replication structures in *sgs1-SIM1-2*Δ mutants exposed to MMS by 2D gel analysis. We focused on the genomic region around the *ARS305* (autonomously replicating sequence 305) origin (Fig. 6A). Origin firing generates intermediates that migrate as a bubble arc containing forks proceeding bidirectionally, large Y molecules resulting from forks migrating outside of the restriction fragment, and specialized X-shaped structures representing HJs (Fig. 6A). Cells were arrested in G1, released into S phase in the presence of MMS, and processed for analysis 3 h after the initial release from G1. In wild-type cells, X-shaped structures appear early during replication and are slowly resolved as cells progress through S phase (data not shown); however, *sgs1*Δ cells accumulate these structures (Fig. 6B; Liberi et al. 2005). Cells carrying the *sgs1-SIM1-2*Δ allele exhibited a clear accumulation of HJs compared with wild-type cells (Fig. 6A). This is consistent with our results showing an impairment of *sgs1-SIM1-2*Δ in recruitment to chromatin upon DNA damage (Fig. 5H), likely due to disrupted interaction with Smc5/6 (Fig. 4C). Similar results were seen in *sgs1-SIM1*Δ (data not shown), which confirms the importance of Smc5–Sgs1 interactions for Sgs1's role in preventing joint molecules at damaged forks. Unlike *sgs1-SIM1-2*Δ, the *sgs1-3KR* allele interacts normally with Smc5/6 (Fig. 5C), is recruited to chromatin (Fig. 5G), and is defective only in SUMO modification (Fig. 5B). We therefore used this allele to test whether Sgs1 SUMOylation, but not its recruitment to chromatin, is also necessary for the resolution of joint molecules or HJs at damaged forks. Similar to *sgs1*Δ, the *sgs1-3KR* mutant accumulated these molecules (Supplemental Figs. S6B, S9), demonstrating that Sgs1 SUMOylation is indeed necessary for the role of STR in preventing these structures. Moreover, the single-site mutant *sgs1-K621R* also exhibited an accumulation of HJs in MMS (data not shown), which indicates that even preventing the SUMOylation of the main SUMO acceptor site has an effect only on Sgs1's function.

Growth assays on medium plates containing MMS showed that SUMO-deficient *sgs1-3KR* cells are only marginally more sensitive to this drug than cells with the Sgs1 wild-type allele (Fig. 6C). We considered the possibility that alternative pathways involved in the resolution of HJs could compensate for the compromised dissolution activity observed in the *sgs1-3KR* genetic background (Fig. 6B). The structure-specific endonuclease Mus81–Mms4 has been shown to resolve HJs at damaged forks in the absence of STR (Ashton et al. 2011). We therefore looked for genetic interactions between Mms4 and *sgs1-3KR.* We found that *mms4Δ sgs1-3KR* cells are significantly more sensitive to MMS than the single mutants *mms4*Δ or *sgs1-3KR*, thus exposing an additive effect between the two alleles (Fig. 6C). A similar genetic interaction was found between the *sgs1-SIM1-2*Δ allele and *mms4*Δ (Fig. 6D).

Our data show that blocking Sgs1 SUMOylation affects its activity at damaged forks (Fig. 6B). We decided to use a recent approach to generate a constitutively SUMOylated form of Sgs1 to investigate its effect on joint molecule accumulation. The approach involved fusion of the SUMO E2-conjugating enzyme Ubc9 to target proteins (Jakobs et al. 2007; Almedawar et al. 2012). Fusion of Ubc9 to the C terminus of Sgs1 (Sgs1-Ubc9) generated an allele of Sgs1 that was constitutively SUMOylated (Supplemental Fig. S10). We did not observe accumulation of joint molecules or HJs in cells expressing wild-type Sgs1 or Sgs1-Ubc9 in *sgs1*Δ genetic background cells (Fig. 6E). Fusion of a catalytically dead Ubc9 protein to Sgs1 also prevented HJ accumulation (Fig. 6E). On the contrary*,* cells expressing a fusion of the Ulp SUMO peptidase domain (UD) of the protease Ulp1 to Sgs1 (which prevented Sgs1 SUMOylation) (Supplemental Fig. S10), but not a catalytically dead domain, accumulated HJs in 2D gels (Fig. 6E). This is fully consistent with our results on *sgs1-3KR* (Fig. 6B).

### Sgs1 SUMOylation reduces crossover frequencies during DSB repair

**Figure 7.**
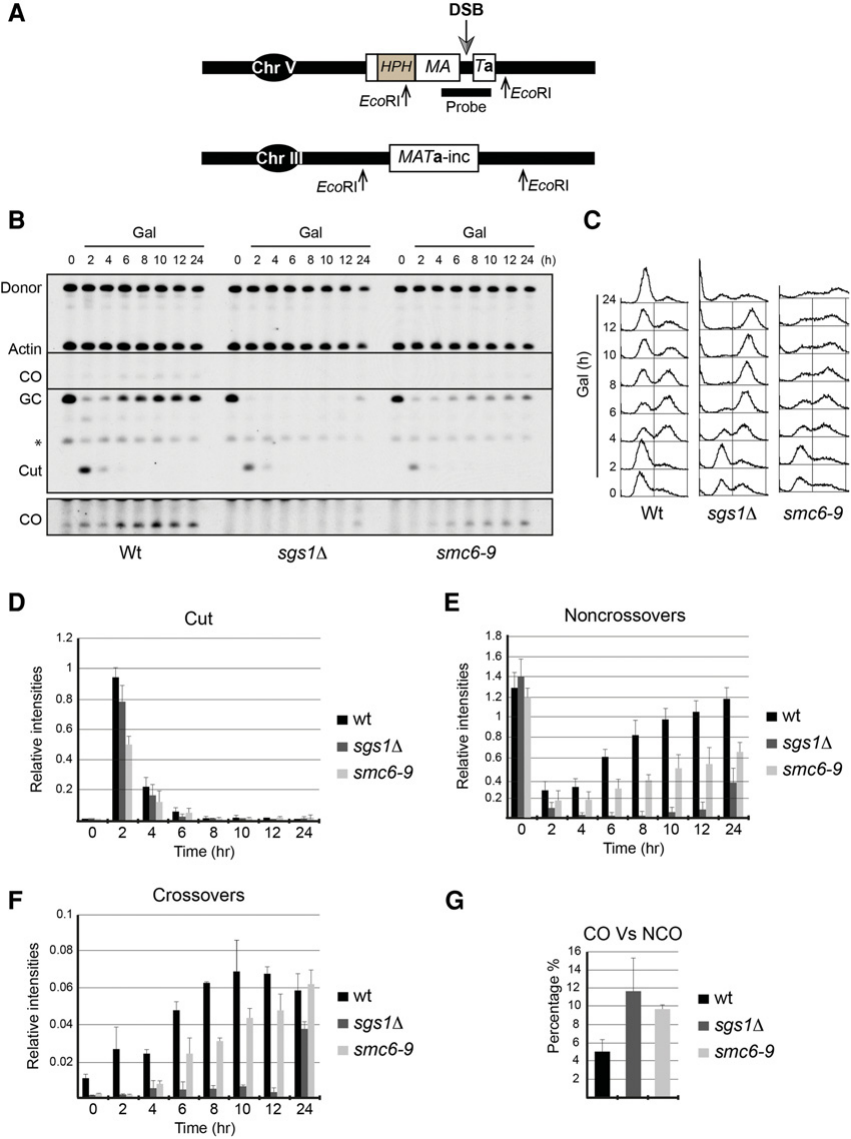
Smc5/6 function reduces crossover frequencies during DSB repair. (*A*) Schematic representation of the interchromosomal recombination assay to measure crossover ratio during DSB repair. (*B*) DSB repair assay of wild-type and *sgs1*Δ and *smc6-9* mutant cells. Cells were grown overnight in YP raffinose and shifted for 30 min to 35°C (semipermissive temperature) to inactivate the *smc6-9* allele. Next, galactose was added at a final concentration of 2% to induce HO expression and provoke a DSB. Cells were collected at the indicated time points and processed. Actin was used as a loading control. A higher exposure of the highlighted region is shown to visualize the crossovers. (*C*) FACS analysis of *B*. (*D*–*G*) Quantification of *B*. Mean values and standard deviations of two independent experiments are shown. Nonsaturated exposures were used for gel quantifications using ImageJ.

In mitotic cells, repair of DSBs by synthesis-dependent strand annealing (SDSA) is rarely associated with crossovers (Ira et al. 2003). Deletion of *SGS1* or *TOP3* has been shown to increase the number of gene conversions with crossovers by threefold (Ira et al. 2003), demonstrating that dHJ dissolution by STR suppresses crossover formation during HR (Ira et al. 2003). Our results demonstrating that Sgs1 SUMOylation plays a role in preventing joint molecules at damaged forks (Fig. 6B) prompted us to investigate a potential role in crossover outcomes during DSB repair in mitotic cells. To this aim, we used an interchromosomal recombination assay previously developed by Haber and colleagues (Ira et al. 2003) that measures crossover ratios during DSB repair. In brief, a DSB within a 2-kb *MATa* sequence, placed on chromosome V, is produced by a galactose-inducible HO endonuclease. Repair of this break proceeds through HR using the *MATa-inc* sequence on chromosome III as a donor (Ira and Haber 2002). Repair occurs only once because a single base pair mutation in the *MATa-inc* donor sequence prevents cleavage by HO after the initial repair event. Repair occurs by gene conversion with or without an accompanying crossover, and the result can be distinguished by the size of fragments using flanking restriction enzymes (Fig. 7A). Crossover frequencies can be accurately calculated based on the density of bands corresponding to noncrossover and crossover products (Ira et al. 2003).

**Figure 8.**
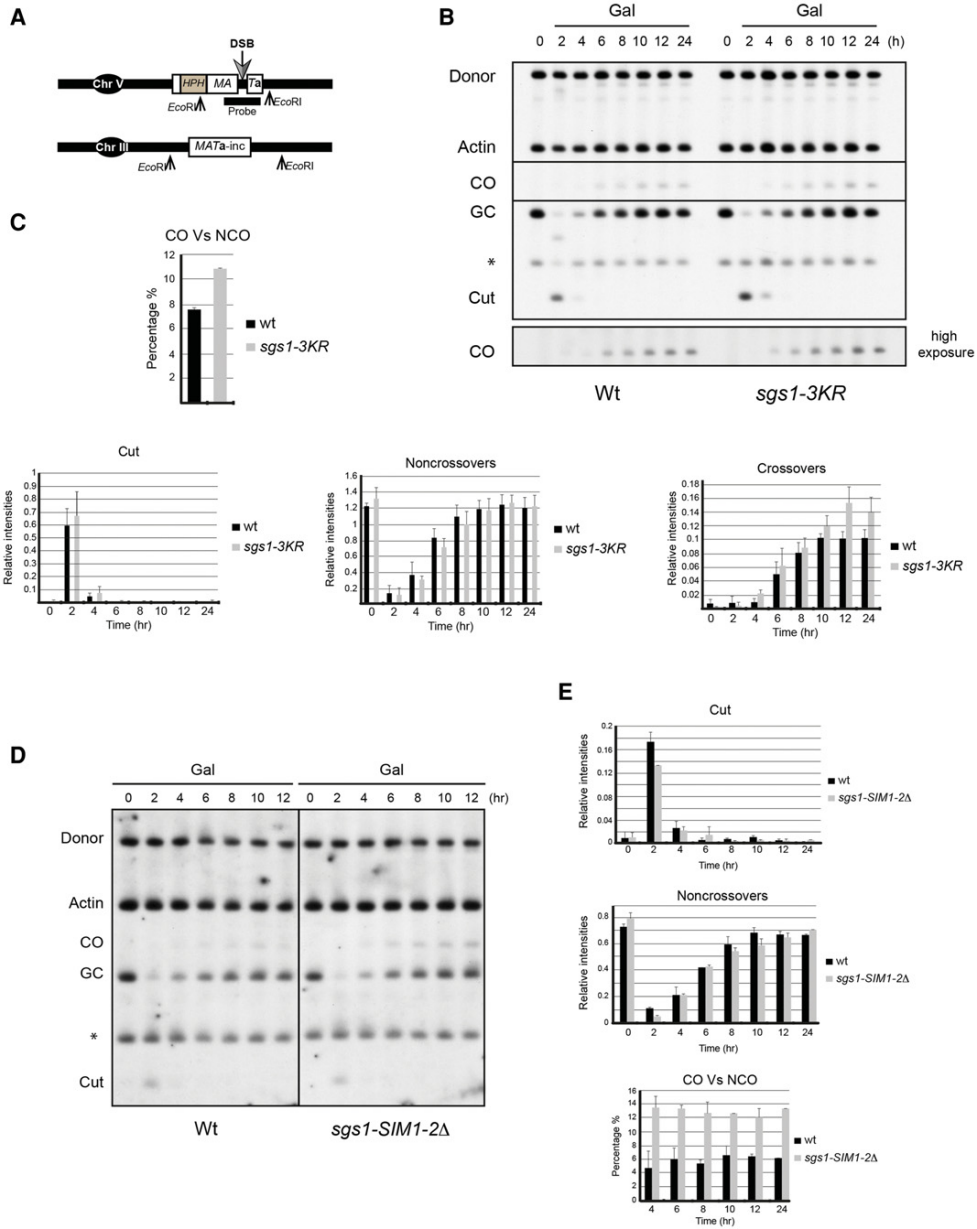
Sgs1 SUMOylation reduces crossover frequencies during DSB repair. (*A*) Schematic representation of the interchromosomal recombination assay to measure crossover ratio during DSB repair. (*B*) DSB repair assay of wild-type and *sgs1-3KR* mutant cells. Cells were grown overnight in YP raffinose. Galactose was added at a final concentration of 2% to induce HO expression and provoke a DSB. Cells were collected at the indicated time points and processed. Actin was used as a loading control. A higher exposure of the highlighted region is shown to visualize the crossovers. (*C*) Quantification of *B*. Mean values and standard deviations of three independent experiments are shown. Nonsaturated exposures were used for gel quantifications using ImageJ. (*D*) DSB repair assay of wild-type and *sgs1-SIM1-2*Δ mutant cells. Cells were grown overnight in YP raffinose. Galactose was added at a final concentration of 2% to induce HO expression and provoke a DSB. Cells were collected at the indicated time points and processed. Actin was used as a loading control. (*E*) Quantification of *D*. Mean values and standard deviations of three independent experiments are shown. Nonsaturated exposures were used for gel quantifications using ImageJ.

First, we tested whether inactivation of Smc5/6 had an effect on crossover frequencies. We used the conditional allele *smc6-9* at the semipermissive temperature of 35°C (since induction of HO by galactose addition is inefficient at 37°C) to probe for Smc5/6 involvement. We used actin as an internal loading control in our blots and analyzed the kinetics of repair in wild-type, *sgs1*Δ, and *sm6-9* cells over a 24-h time period (Fig. 7B–G). Formation of the induced DSB was efficient and comparable in the three strain backgrounds (Fig. 7D). In wild-type cells, gene conversions without crossover appeared to increase significantly at 6–8 h after DSB formation (Fig. 7E), in contrast to *sgs1*Δ and *smc6-9* cells, where noncrossover products were significantly reduced, particularly in *sgs1*Δ (Fig. 7E). We were surprised by these results; crucially, we were able to rescue the defects in noncrossover formation when we introduced a wild-type copy of *SGS1* in *sgs1*Δ (Fig. 8), demonstrating that this is a direct effect of Sgs1's absence. Crossover products appeared with timing similar to that of noncrossovers in wild-type cells and were slower and marginally reduced in *sgs1*Δ and *smc6-9* cells (Fig. 7F). When we calculated the percentage of gene conversions with associated crossovers over the total repair observed (i.e., taking into consideration both crossover and low-frequency noncrossover products), gene conversions in wild-type cells were associated with crossovers in 5% of the cases, while this was increased in *sgs1*Δ to 11%, as previously reported, (Ira et al. 2003) and in *smc6-9* to 9.5% (Fig. 7G). Based on these results, we conclude that, like Sgs1, Smc5/6 function suppresses crossover formation during DSB repair.

Next, we investigated whether this effect occurs through Smc5/6-dependent SUMOylation of Sgs1. To this aim, we tested *sgs1-3KR* in the interchromosomal recombination assay described (Fig. 8A–C). Formation of the induced DSB was equally efficient in the two strain backgrounds (Fig. 8C). Unlike *sgs1*Δ and *smc6-9* cells, *sgs1-3KR* cells showed kinetics of gene conversion without associated crossovers very similar to that of wild-type cells (Fig. 8C), suggesting that this function is not affected by the lack of Sgs1 SUMOylation. However, like *sgs1*Δ and *smc6-9* cells, *sgs1-3KR* cells exhibited an increase over wild-type cells in the percentage of gene conversions with associated crossovers over total repair (Fig. 8B,C). We obtained similar results when we tested the *sgs1-SIM1-2*Δ allele in the interchromosomal recombination assay (Fig. 8D,E). These results demonstrate that Smc5/6-dependent recruitment and SUMOylation of Sgs1 contribute to the suppression of crossover formation during DSB repair normally observed in mitotic cells.

### Sgs1's 5′-to-3′ resection function requires its SUMOylation

**Figure 9.**
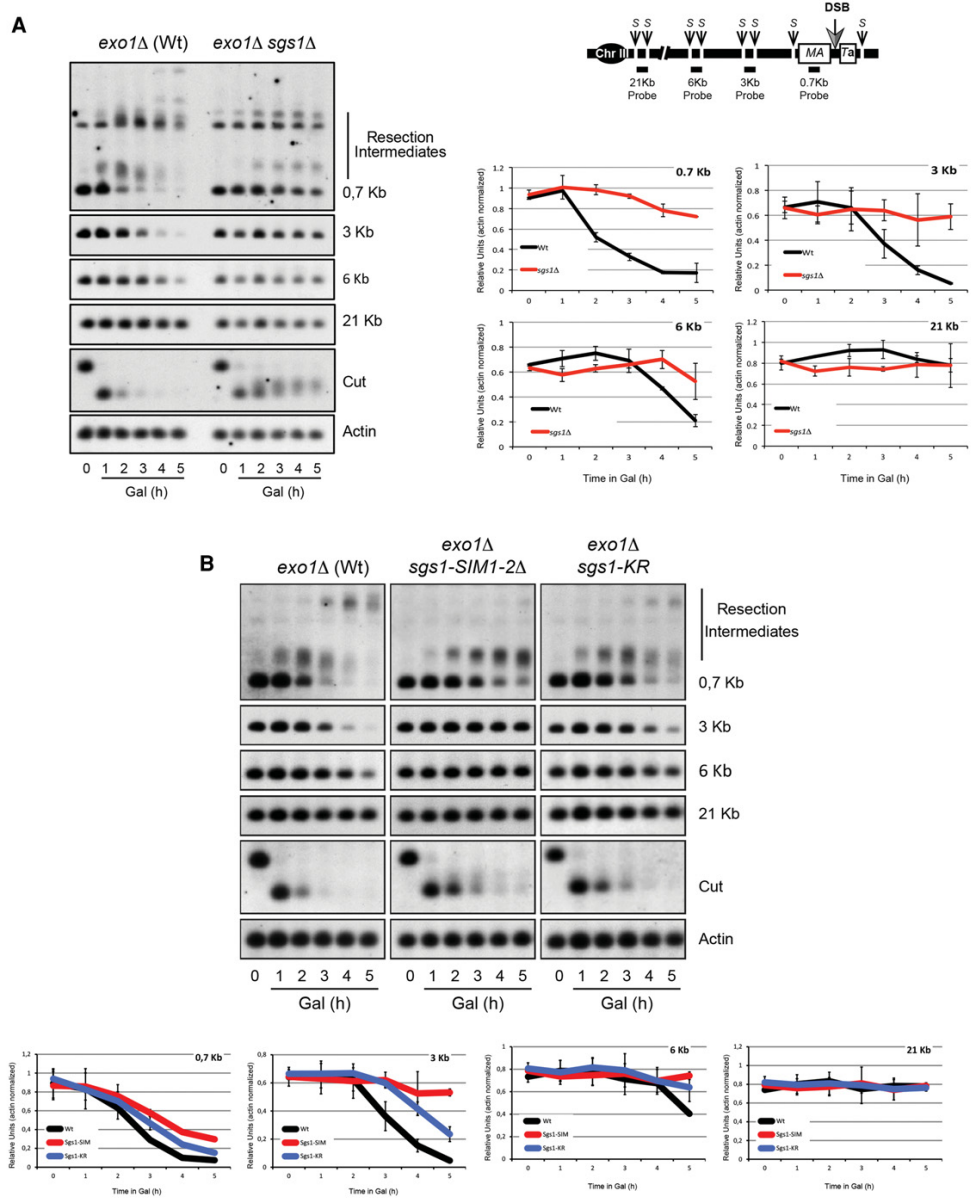
Sgs1 SUMOylation is required for its 5′ end resection activity. (*A*) Schematic representation of the region analyzed. Four probes were used at different distances from the DSB. (S) StyI restriction sites used to digest genomic DNA. Resection assay comparing wild type (*exo1*Δ) and *sgs1*Δ (*exo1Δ sgs1*Δ). Cells were grown on YP raffinose overnight and arrested in G1 with α factor. Once arrested, cells were released from the G1 arrest into YP raffinose. Next, galactose at a final concentration of 2% was added to induce the expression of the endonuclease HO and induce a DSB. Samples were taken every hour for 5 h and analyzed by Southern blot. Southern blot quantifications. Band intensities were normalized with actin as a loading control. Mean values and standard deviations of two independent experiments are shown. Nonsaturated exposures were used for gel quantifications using ImageJ. Note that *sgs1*Δ mutants show a complete impairment in 5′ end resection. (*B*) Resection assay comparing wild-type (*exo1*Δ) and *sgs1-SIM1-2*Δ (*exo1Δ sgs1-SIM1-2*Δ) and *sgs1-KR* (*exo1Δ sgs1-KR)* mutant cells. Cells were grown and analyzed as in *A*. Note that *sgs1-KR* and *sgs1-SIM1-2*Δ mutants show a severe impairment in 5′ end resection.

Our results demonstrate that Sgs1 interaction with Smc5/6 is important to recruit the helicase to chromatin when cells encounter DNA damage (Fig. 5F) and that Smc5/6-dependent SUMOylation of Sgs1 is required for Sgs1's recombination functions (Figs. 6B, 8). Both Smc5/6 and Sgs1 are recruited to DNA DSBs (De Piccoli et al. 2006; Zhu et al. 2008b). Sgs1 together with Top3–Rmi1 and the endonuclease Dna2 plays a role in DNA end resection of DSBs (Gravel et al. 2008; Mimitou and Symington 2008; Zhu et al. 2008b). We therefore sought to test whether Smc5/6 also regulates the function of Sgs1 in end resection via SUMOylation. To this aim, we analyzed how breaks are resected in both *sgs1-SIM1-2*Δ cells, where the interaction between Smc5/6 and STR is impaired (Fig. 4C), and *sgs1-KR* cells, where Sgs1 SUMOylation but not its interaction with Smc5/6 is defective (Fig. 5C). We used a strain that has a single HO endonuclease recognition site at the *MAT* locus on chromosome III. The DSB can be induced at the *MAT* locus by controlled expression of the HO endonuclease but cannot be repaired because the donor sequences *HMR* and *HML* are deleted. We used a genetic background deficient for Exo1, a nuclease that works in an end resection pathway parallel to Sgs1 (Gravel et al. 2008; Mimitou and Symington 2008; Zhu et al. 2008b), to observe resection events that are dependent on Sgs1 function. Following synchronous HO-induced cleavage, we monitored resection within 20 kb of the break site using a set of probes specific for sequences at different distances (Fig. 9A,B). We measured band intensities corresponding to the probes over a 5-h period. In *exo1*Δ cells where wild-type Sgs1 resection activity is present, we detected resection up to 6 kb away from the break, with complete resection in regions 3 kb away by 4 h (Fig. 9A,B). Resection was completely abolished in *exo1Δ sgs1*Δ cells (Fig. 9A). Importantly, resection was significantly reduced in *exo1Δ sgs1-SIM1-2*Δ cells, with only limited detection 700 base pairs (bp) away and no appreciable resection at 3 kb by 5 h (Fig. 9B). This finding demonstrates that preventing Sgs1's interaction with Smc5/6 severely compromises Sgs1-dependent resection. Similarly to *exo1Δ sgs1-SIM1-2*Δ, *exo1Δ sgs1-KR* cells exhibited a decrease in resection in this assay (Fig. 9B), demonstrating that, in addition to the interaction with Smc5/6, Sgs1 needs to be SUMOylated by Smc5/6 to fully carry out its role in 5′ strand resection.

## Discussion

Both the yeast Sgs1 helicase and its mammalian ortholog, BLM, carry out numerous functions in the repair of DSBs and replication forks and thus represent a key node to maintain genome integrity (Larsen and Hickson 2013). However, these helicases can perform both pro- and anti-recombinogenic tasks, often acting on similar substrates. Thus, their activity must necessarily be tightly controlled to prevent inappropriate events with detrimental effects for the genome. Indeed, recent studies have highlighted roles for post-translational modifications (PTMs) of BLM, including phosphorylation, SUMOylation, and ubiquitination, to mediate fine-tuning of its function (for review, see Bohm and Bernstein 2014).

**Figure 10.**
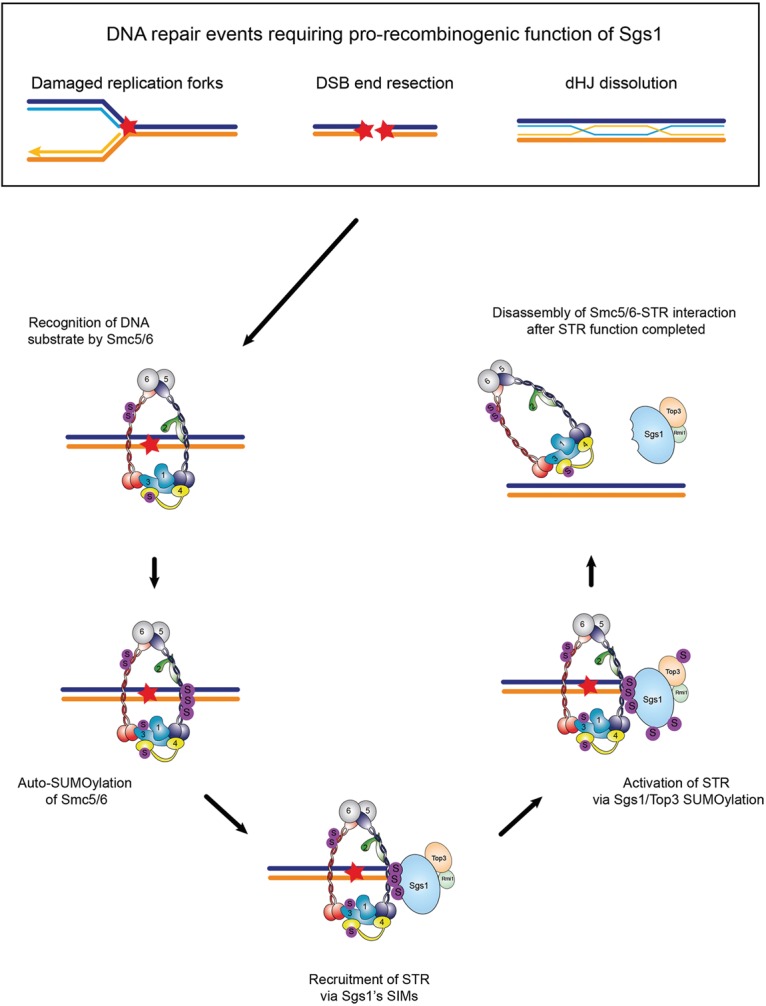
SUMOylation-dependent recruitment and activation of STR by Smc5/6. Schematic representation of the regulation of Sgs1's prorecombinogenic functions by Smc5/6. Upon DNA damage requiring STR function at different steps of HR repair, including DSB end resection, dHJ dissolution, and fork rescue, we propose that Smc5/6 is able to recognize suitable DNA substrates for Sgs1. Binding of Smc5/6 to DNA substrate structures (“Recognition of DNA substrate by Smc5/6”) leads to ATP-dependent remodeling of Smc5/6 and activation of the SUMO E3 ligase Mms21, as demonstrated earlier (Bermudez-Lopez et al. 2015). Mms21 then SUMOylates several subunits of Smc5/6 (“Auto-SUMOylation of Smc5/6”). STR recruitment to these sites occurs via the recognition of SUMOylated Smc5/6 through the SIM domains of Sgs1 (“Recruitment of STR via Sgs1's SIMs”). Recruited STR is then subject to Mms21-dependent SUMOylation that results in the activation of STR function (“Activation of STR via Sgs1/Top3 SUMOylation”). Upon completion of repair, the Smc5/6–STR interaction is disassembled (“Disassembly of Smc5/6–STR interaction after STR function completed”). Therefore, Smc5/6 is proposed as a central regulator of Sgs1 necessary to switch and regulate Sgs1's prorecombinogenic functions.

Both BLM and Sgs1 are regulated by PTMs, including SUMOylation (Bohm and Bernstein 2014), and some of these modifications have been linked to changes in the localization in response to different types of DNA damage (Eladad et al. 2005; Ouyang et al. 2009; Bohm et al. 2015). However, no reports to date have explained how these modifications lead to the altered localization and whether they affect the intrinsic function of this RecQ helicase. We show here that the Smc5/6 complex regulates a wide range of prorecombinogenic activities of Sgs1, including DNA end resection and dHJ resolution during replication fork and DSB repair. We demonstrate that Smc5/6 regulation of Sgs1 function operates at two levels: First, Smc5/6 is responsible for recruitment of Sgs1 to chromatin templates and second, once recruited, Smc5/6-dependent SUMOylation of Sgs1 is necessary for the activation of Sgs1's prorecombinogenic functions (Fig. 10). Our data offer a mechanistic view of the process by which Sgs1 is recruited to sites of DNA damage that require prorecombination activities and how it becomes licensed at these sites through PTMs. We and others have shown previously that Smc5/6 is recruited to sites of DNA damage, including DSBs (De Piccoli et al. 2006; Lindroos et al. 2006; Potts et al. 2006). We found that, upon DNA damage that requires HR-dependent repair, several subunits within the Smc5/6 complex, including Smc5, Smc6, Nse3, and Nse4, are hyper-SUMOylated by Mms21 (Fig. 1B). Therefore, it is likely that Smc5/6 complexes recruited to damaged sites are hyper-SUMOylated. We found that Sgs1 is capable of identifying such sites through the recognition of SUMOylated Smc5/6 complexes due to the presence of two SIMs, which we show to be specific for the recognition of SUMOylated Smc5/6 (Fig. 4). The ability to recognize SUMOylation on different proteins to regulate its subnuclear localization has been also described for Sgs1's homolog, BLM, which is recruited to PML bodies through its SIM domains (Zhu et al. 2008a), although the SUMOylated protein that attracts BLM to PML bodies is unknown. It is noteworthy that PML bodies that contain DNA in ALT cells, also called APBs, not only contain Smc5/6 (Potts and Yu 2007) but also require Smc5/6-dependent SUMOylation of the shelterin complex, an event thought to mediate the recruitment of telomere repeats to APBs (Potts and Yu 2007). Moreover, BLM has been implicated in ALT pathways, localizes to APBs, and interacts with the shelterin subunits TRF1 and TRF2 (Stavropoulos et al. 2002; Lillard-Wetherell et al. 2004; Zimmermann et al. 2014), and its overexpression promotes increased telomeric HR and longer telomeres (Stavropoulos et al. 2002). It is tempting to speculate that Smc5/6 might promote telomeric recombination in ALT cells by coordinating recruitment of telomeres and BLM to APBs and activating the prorecombinogetic role of BLM in a manner analogous to what we demonstrated here for Sgs1.

Sgs1 and BLM also have anti-recombinogenic roles, such as when replication forks stall as a consequence of HU exposure (Cobb et al. 2003; Sengupta et al. 2003). Sgs1 is required for the stabilization of the DNA polymerases at HU-stalled forks (Bjergbaek et al. 2005; Cobb et al. 2005). Recent work has shown that situations in which Sgs1 prorecombinogenic functions are required, such as exposure to MMS, correlate with an increase in the number of cells showing Sgs1 nuclear foci (Bohm et al. 2015), while anti-recombinogetic roles are associated with a reduction (Bohm et al. 2015). The reduction of Sgs1 foci required the checkpoint kinase Mec1 and the SUMO targeted ubiquitin ligases (STUbLs) Slx5/Slx8 (Bohm et al. 2015). Our data show that exposure to HU does not trigger increased levels of Sgs1 SUMOylation (Fig. 2E) unless effector kinases of the DNA damage checkpoint are absent (Fig. 2F). This is in complete agreement with a function for STUbLs in removing SUMOylated Sgs1 in HU arrests (Bohm et al. 2015). In the absence of checkpoint kinases (Mec1 or Rad53), stalled forks collapse (Lopes et al. 2001), and this is likely to trigger a switch in Sgs1's roles to prorecombinogetic mode, as HR then becomes necessary for repair of these forks. Under such conditions, Sgs1 SUMOylation (Fig. 2F) and an increase in the number of Sgs1 foci are observed (Bohm et al. 2015).

Interestingly, analysis of the SUMO-compromised allele *Sgs1-K621R* demonstrated that this mutant is still able to form foci during an untreated S phase (Bohm et al. 2015). Our results demonstrate that SUMOylation of Sgs1 is necessary for its activation but not its localization to chromatin (Fig. 5G) or interaction with Smc5/6 (Fig. 5C); instead, these two properties are mediated by Sgs1's SIM domains through recognition of SUMOylated Smc5/6 (Fig. 5H).

It has been proposed that Mms21 and Sgs1 work in concert to resolve the recombination structures formed during replication in the presence of DNA damage (Branzei et al. 2006). Here we show that a two-step regulation of Sgs1 by Smc5/6 generates a complex regulation where a combination of SUMO–SIM and SUMO modification events is involved.

Previous reports identified Lys621 in Sgs1 as the major SUMO acceptor site (Lu et al. 2010). *Sgs1-K621R* mutants were shown to be defective in telomere–telomere recombination and therefore were defective in the production of type II survivors in the absence of telomerase (Lu et al. 2010). Interestingly, these are thought to be homologous to mammalian ALT pathways for telomere maintenance (Teng and Zakian 1999), which involve Smc5/6 function (Potts and Yu 2007). Besides a role in telomere recombination, *Sgs1-K621R* mutants were reported to display no other chromosomal HR functions, as no sensitivity to MMS plates was observed despite the fact that Sgs1 SUMOylation occurs specifically in response to treatment with this DNA-damaging agent (Lu et al. 2010). Here we also observed a minor sensitivity of *Sgs1-SIM*Δ and *Sgs1-3KR* mutants to MMS, but our data suggest that this is due to the presence of redundant pathways that can compensate for the prorecombinogetic roles of Sgs1, such as endonuclease resolution of dHJs (Fig. 6C) or DSB resection by the Exo1 nuclease, rather than being caused by the lack of involvement of Sgs1 SUMOylation in these chromosomal HR functions. Our findings show that the effect of SUMOylation in the HR functions of Sgs1 is as prominent as in BLM (Eladad et al. 2005), demonstrating that Sgs1 is a good model to study BLM function.

While the function of Sgs1 during HR has been the subject of many studies, it is not well understood how Sgs1 identifies DNA substrates during its activities. Here we demonstrated the mechanisms that guide and regulate Sgs1 (and STR) to its DNA substrates. We show that the Smc5/6 complex is a key regulator of Sgs1 prorecombinogenic functions. We show that Smc5/6 auto-SUMOylation guides Sgs1 to chromatin and demonstrated that this relies on the ability of Sgs1 to specifically recognize SUMOylated Smc5/6 through SIM domains. We then found that Sgs1 and Top3 are subject to SUMO modification in the hands of the Smc5/6 SUMO E3 ligase Mms21. Moreover, this modification is required for the correct function of STR in several steps of HR, from DNA end resection to dHJ dissolution (Fig. 10). Through such roles, Smc5/6 and Sgs1 SUMOylation are critical to ensure the suppression of crossover outcomes during the DSB repair and the correct processing of HR intermediates appearing at replication forks during their repair—both critical requirements for genome stability.

Understanding the molecular mechanisms of Sgs1/BLM function is very relevant to human health. Here we bring a new key player to the area, Smc5/6, and, importantly, demonstrate that this complex is a master regulator that switches Sgs1 toward HR functions. Based on the role of BLM in genome stability and ALT activation in tumors, fully understanding the role of Sgs1/BLM and its direct regulators as well as the specific effect of PTMs on its function could potentially provide novel therapeutic targets and biomarkers. We hope that the findings reported here inform future exploration of the important roles of Smc5/6 and STR in maintaining genome stability.

## Materials and methods

### Yeast strains and plasmids

A list of strains and plasmids used in this study is in Supplemental Tables S1 and S2. Epitope tagging of genes and deletions were performed as described in Goldstein and McCusker (1999) and Janke et al. (2004). *Sgs1-K621R*, *sgs1-3KR*, and *sgs1-SIM1-2*Δ alleles were inserted at the endogenous *SGS1* locus. Plasmids were synthetized by GeneCust (Luxembourg). They were amplified in *Escherichia coli*, linearized, and transformed into yeast strains.

### Yeast growth conditions

Yeast cells were grown in yeast extract peptone (YEP) or minimum Complete medium (SC) to select for plasmid auxotrophies plus the indicated carbon source at 2% final concentration. To induce DNA damage, cells were treated with MMS, HU, or phleomycin at different final concentrations depending on the experiment (see the figure legends).

### Cell cycle synchronizations

Yeast cells were grown in YPD at 25°C except when otherwise stated. To synchronize cells in G1, exponentially growing cultures were treated with α factor (Insight Biotechnology). For *BAR1*^+^ strains, the final concentration used was 10^−6^ M and 10^−8^ M for *bar1*Δ cells. They were maintained in the presence of α factor until >95% had been arrested in G1. The release from α factor was conducted by washing cells twice with prewarmed medium and resuspending them in medium containing 0.1 mg/mL protease from *Streptomyces griceus* (Sigma, pronase). To synchronize cells in metaphase, nocodazole (from Sigma) was used at a final concentration of 15 μg/mL in the presence of 1% DMSO from 1.5 mg/mL stock. For prolonged metaphase arrests, the concentration of nocodazole was raised to 22.5 μg/mL.

### Yeast growth test analysis

Cells were inoculated in liquid medium at 25°C from freshly streaked plates until the culture reached mid-log phase. Next, 10-fold serial dilutions from a culture at OD_600_ ≈ 1.0 were spotted as 2-µL drops onto solid medium, incubated for 3 d at 25°C, and then photographed.

### SUMO pull-down assays

Pull-down analyses were performed essentially as described (Bermudez-Lopez et al. 2015). Cells were denatured during harvesting and prior to snap freezing by sequential resuspension of yeast cells in 12% TCA and 1 M Tris-HCl (pH 8.0). Cells were mechanically broken in 8 M urea buffer and incubated with Ni-NTA beads in the presence of 20 mM imidazole for 2 h at 4°C. Bound proteins were eluted with SDS-PAGE loading buffer. In all cases, SUMO pull-down assays were loaded in SDS-PAGE gels next to protein extracts to confirm the slower mobility of SUMO conjugates.

### Coimmunoprecipitation analysis

Coimmunoprecipitation analyses were performed as previously described in Bermudez-Lopez et al. (2015). Cells were mechanically broken in 50 mM HEPES, 150 mM KCl, 1.5 mM MgCl_2_, 0.5 mM DTT, and 0.5% Triton X-100 (pH 7.5) supplemented with Complete protease inhibitor cocktail tablets (from Roche). Cell extracts were incubated with beads for 2 h at 4°C. Myc-tagged proteins were immunoprecipitated using anti-Myc antibodies (Roche, 9E10) coupled to protein G Dynabeads (Invitrogen). Bound proteins were eluted with SDS-PAGE loading buffer and analyzed by Western blot.

### Chromatin-binding assays

Chromatin-binding assays were performed as previously described in Liang and Stillman (1997). Cells were incubated in 100 mM KH_2_PO_4_/K_2_HPO_4_ (pH 9.4), 10 mM DTT, and 0.1% sodium azide for 10 min. Next, cells were spheroplasted in 100 mM KH_2_PO_4_/K_2_HPO_4_ (pH 7.5), 0.6 M sorbitol, and 10 mM DTT containing zymolase 100T for 10 min at 37°C. Spheroplasts were lysed with Triton X-100 at a final concentration of 0.25% in 100 mM KCl, 50 mM HEPES-KOH (pH 7.5), and 2.5 mM MgCl_2_ supplemented with Complete protease inhibitor cocktail tablets (Roche). The suspension was split into two tubes (one for whole-cell extract [WCE] and the other for chromatin pellet [CP]). Afterward, the lysate was underlayered with 30% sucrose and spun down at 12,000 rpm for 10 min at 4°C. After washing the pellet, an equal volume of SDS loading buffer was added to each fraction and analyzed by Western blot. The abundance of Sgs1 on chromatin was normalized with histone H3 as an internal loading control in our blots. Mean values and standard deviations of two independent experiments are shown. Nonsaturated exposures were used for gel quantifications using ImageJ.

### Western blot

All proteins were resolved in 7.5% SDS-PAGE gels, except Sgs1 (6% SDS-PAGE gel), Rmi1 (10% SDS-PAGE gel), and histone H3 (15% SDS-PAGE gel). Proteins were transferred to polyvinylidene fluoride (PVDF) membranes using the TE70X semidry blotter system (Hoefer). The antibodies used were anti-HA (Roche, 3F10), anti-H3 (Abcam, ab1791), anti-myc (Roche, 9E10), anti-PGK1 (Thermo Scientific, 459250), anti-RFA (Agrisera, AS07 214), and anti-SMT3 (Abcam, ab14405). Blots were incubated with the ECL Prime Western blotting detection reagent (GE Healthcare) followed by exposure to high-performance chemiluminescence films (Amersham Hyperfilm ECL, GE Healthcare) to detect the signal.

### 2D gel electrophoresis

DNA extraction was performed in the presence of hexadecyltrimethylammonium bromide (CTAB) to stabilize branched DNA intermediates as described previously in Mankouri et al. (2007). After purification, DNA concentration was determined, and DNA was digested with HindIII and EcoRV and separated by electrophoresis. 2D gel electrophoresis was carried out using the following conditions: first dimension: 0.4% agarose gel in 1× TBE run at 0.6 V/cm for 24 h at room temperature; second dimension: 1% agarose gel in TBE buffer supplemented with 0.3 μg/mL ethidium bromide at 3 V/cm for 11 h at 4°C. Finally, DNA molecules present in the *ARS305* were detected by Southern blot.

### DSB repair assays: crossover formation and end resection assays

Frozen pellets were resuspended in lysis buffer (50 mM Tris-HCl, 100 mM NaCl, 10 mM EDTA, 1% SDS at pH 8.0). The cell wall was digested with 40 U of lyticase (Sigma) and 1% β-mercaptoethanol at 37°C. Next, DNA was extracted with phenol/chloroform/isoamylalcohol (25:24:1), precipitated with 2 vol of 100% ethanol, and washed with 70% ethanol before being resolubilized in TE buffer. Genomic DNA was digested with EcoRI or StyI for the crossover assay and resection assay, respectively. Digested genomic DNA was loaded onto a 1% agarose gel and run at 35 V for 12 h (resection assay) or 24 h (crossover assay). Finally, DNA fragments were detected by Southern blot.

## Supplementary Material

Supplemental Material
