# Supplementary material for: Sgs1's roles in DNA end resection, HJ dissolution, and crossover suppression require a two-step SUMO regulation dependent on Smc5/6
Source: Genes Dev. 2016 Jun 1;30(11):1339–56. doi: 10.1101/gad.278275.116 (PMC4911932; doi:10.1101/gad.278275.116)
Supplement: Supplemental Material [file supp_30_11_1339__index.html]

Supplemental Material 

# Sgs1's roles in DNA end resection, HJ dissolution, and crossover suppression require a two-step SUMO regulation dependent on Smc5/6

## Supplemental Material

**Files in this Data Supplement:**

- Supplemental\_Figure\_S1.pdf
- Supplemental\_Figure\_S10.pdf
- Supplemental\_Figure\_S2.pdf
- Supplemental\_Figure\_S3.pdf
- Supplemental\_Figure\_S4.pdf
- Supplemental\_Figure\_S5.pdf
- Supplemental\_Figure\_S6.pdf
- Supplemental\_Figure\_S7.pdf
- Supplemental\_Figure\_S8.pdf
- Supplemental\_Figure\_S9.pdf
- Supplemental\_Material.docx
