# Supplementary figures and images for: Sgs1's roles in DNA end resection, HJ dissolution, and crossover suppression require a two-step SUMO regulation dependent on Smc5/6
Source: Genes Dev. 2016 Jun 1;30(11):1339–56. doi: 10.1101/gad.278275.116 (PMC4911932; doi:10.1101/gad.278275.116)

Figure S1

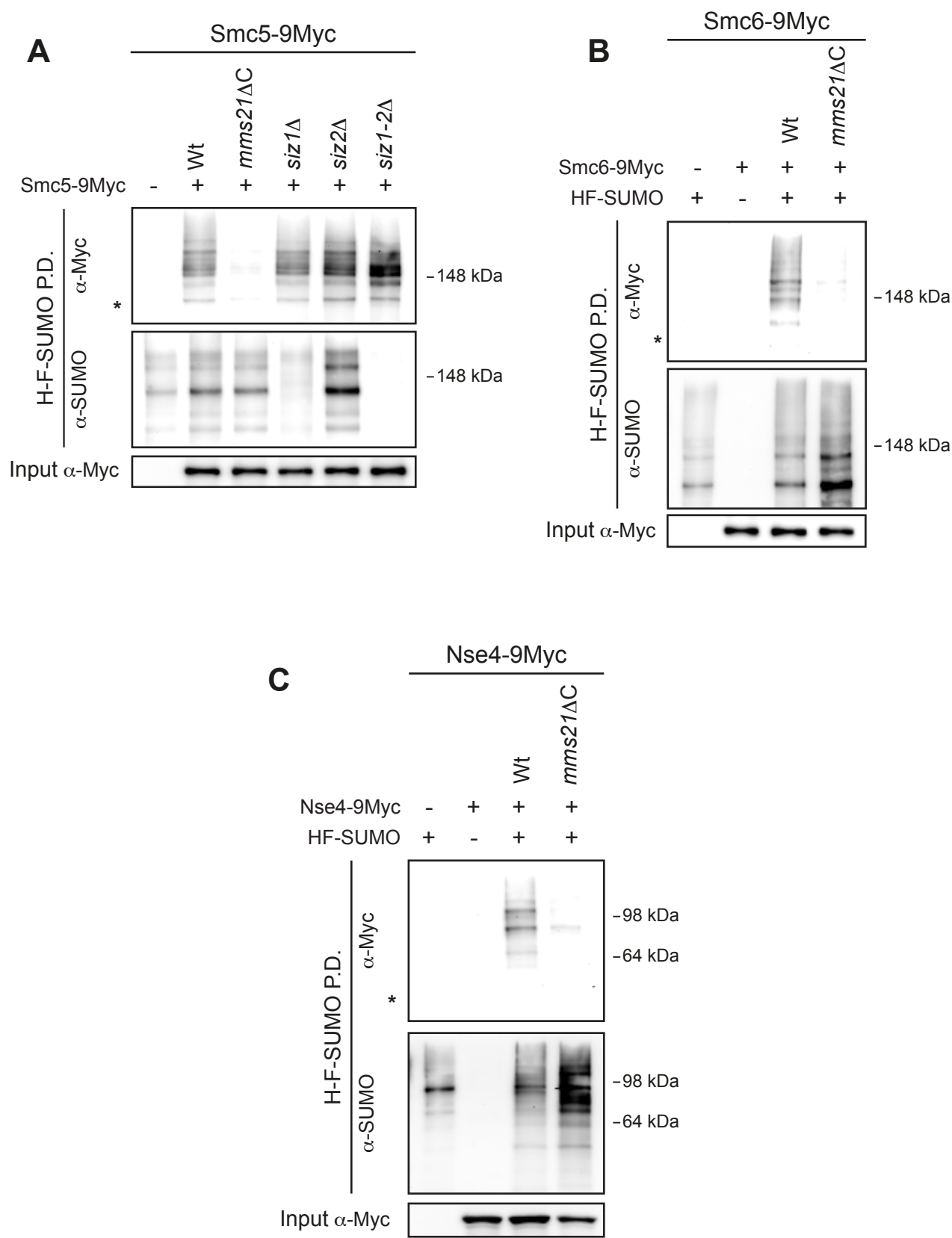

Supplement: Supplemental Material [file supp_30.11.1339_Supplemental_Figure_S1.pdf]

Figure S10

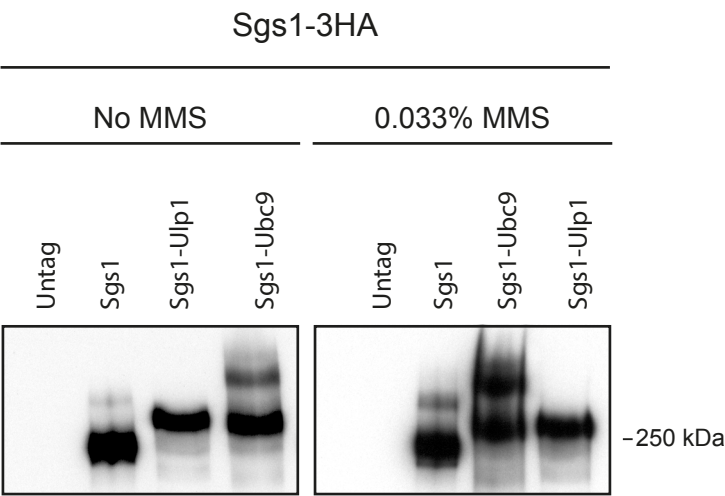

Supplement: Supplemental Material [file supp_30.11.1339_Supplemental_Figure_S10.pdf]

Figure S2

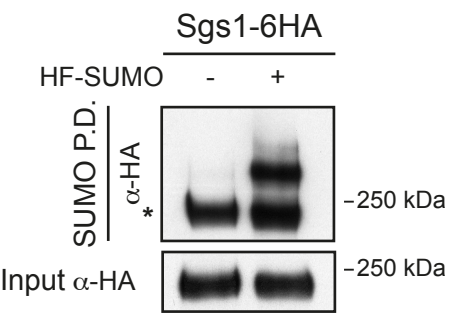

Supplement: Supplemental Material [file supp_30.11.1339_Supplemental_Figure_S2.pdf]

Figure S3

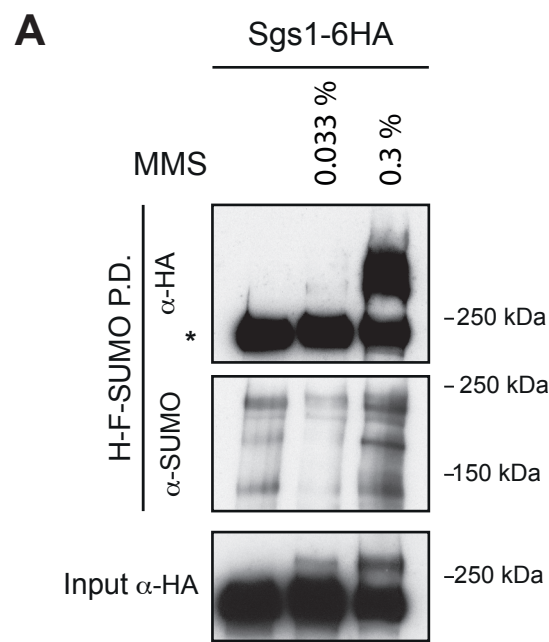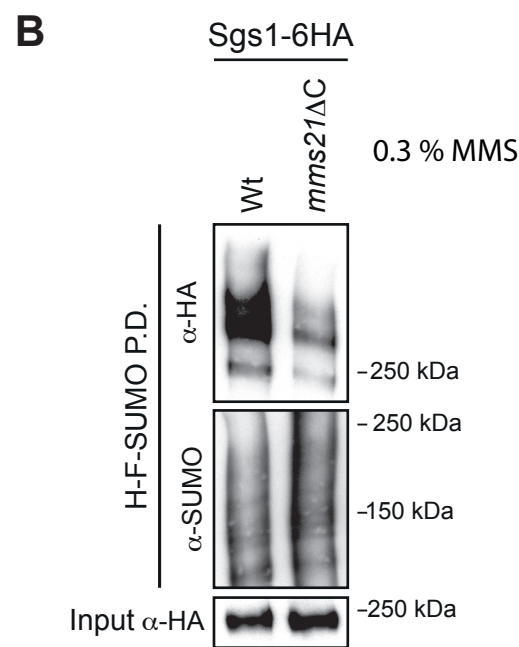

Supplement: Supplemental Material [file supp_30.11.1339_Supplemental_Figure_S3.pdf]

Figure S4

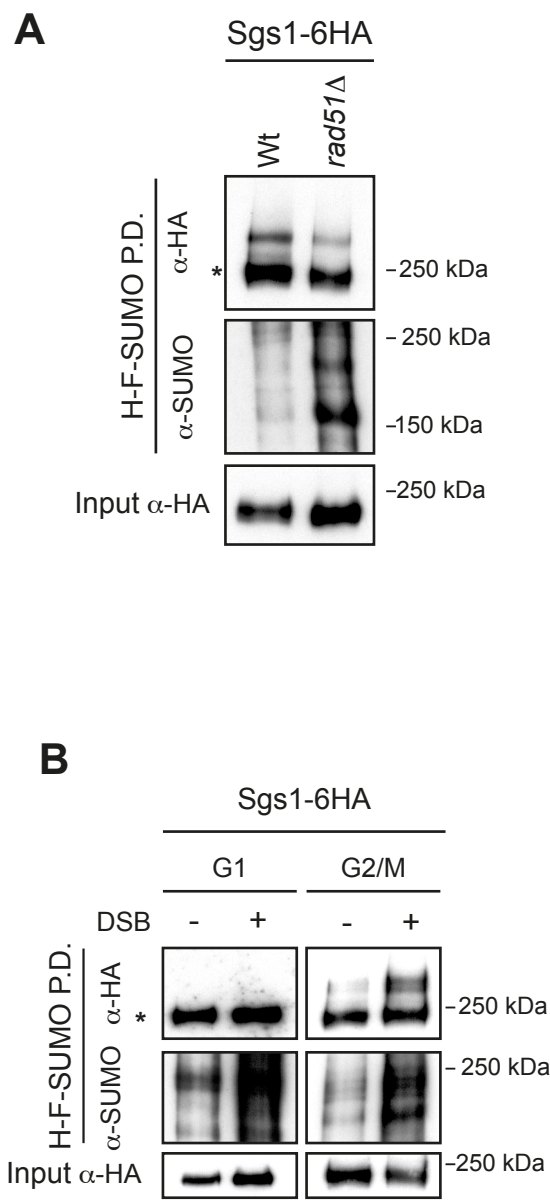

Supplement: Supplemental Material [file supp_30.11.1339_Supplemental_Figure_S4.pdf]

Figure S5

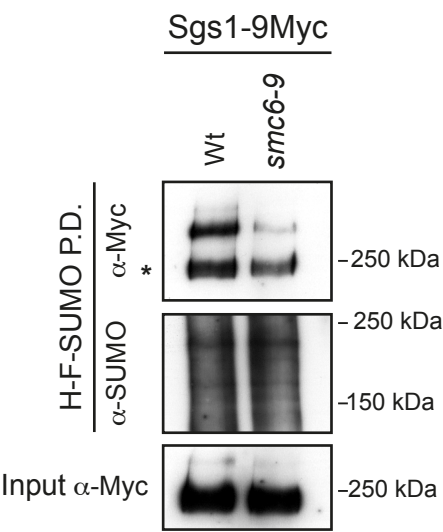

Supplement: Supplemental Material [file supp_30.11.1339_Supplemental_Figure_S5.pdf]

Figure S6

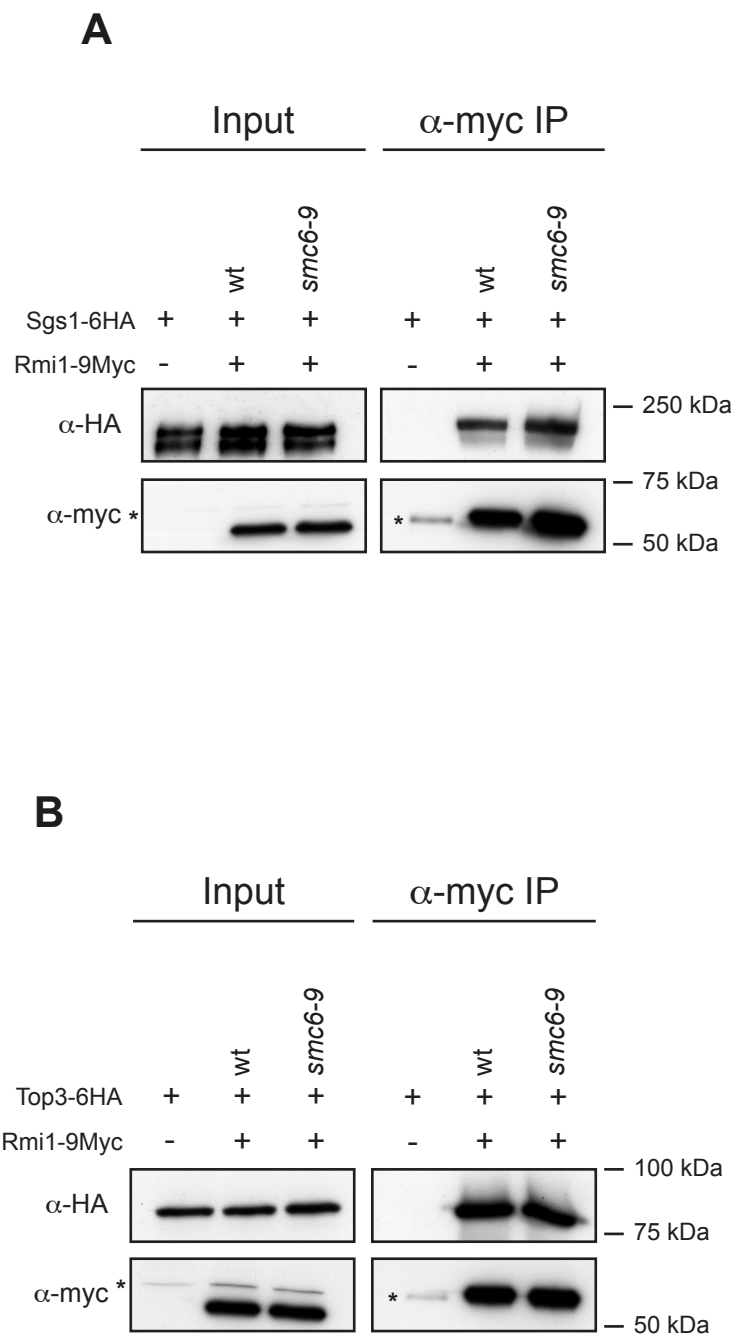

Supplement: Supplemental Material [file supp_30.11.1339_Supplemental_Figure_S6.pdf]

Figure S7

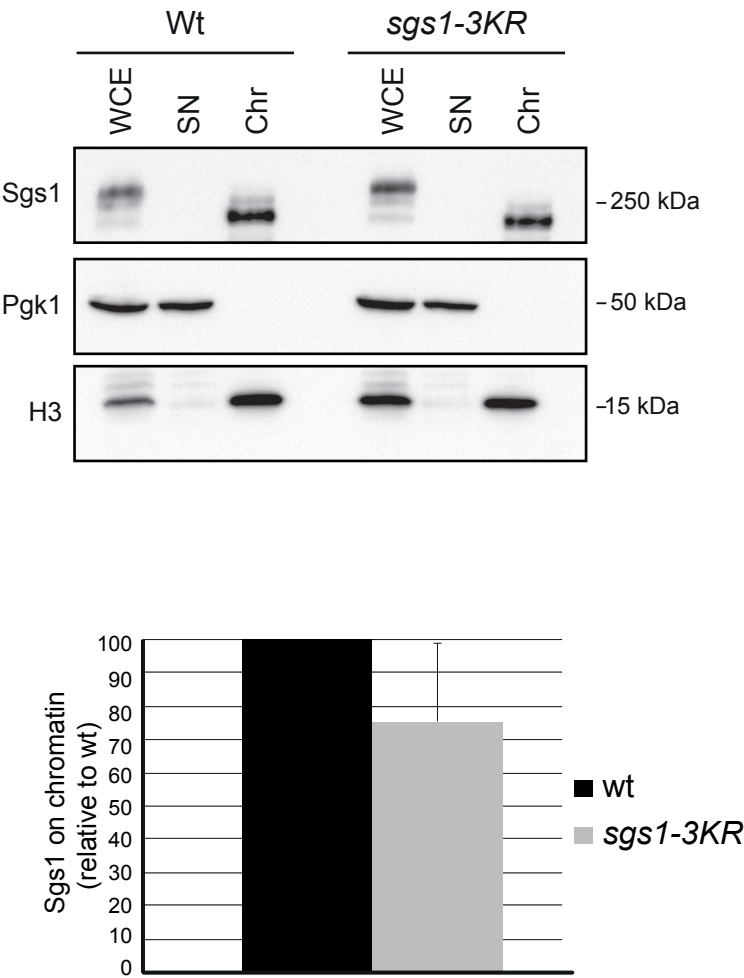

Supplement: Supplemental Material [file supp_30.11.1339_Supplemental_Figure_S7.pdf]

Figure S8

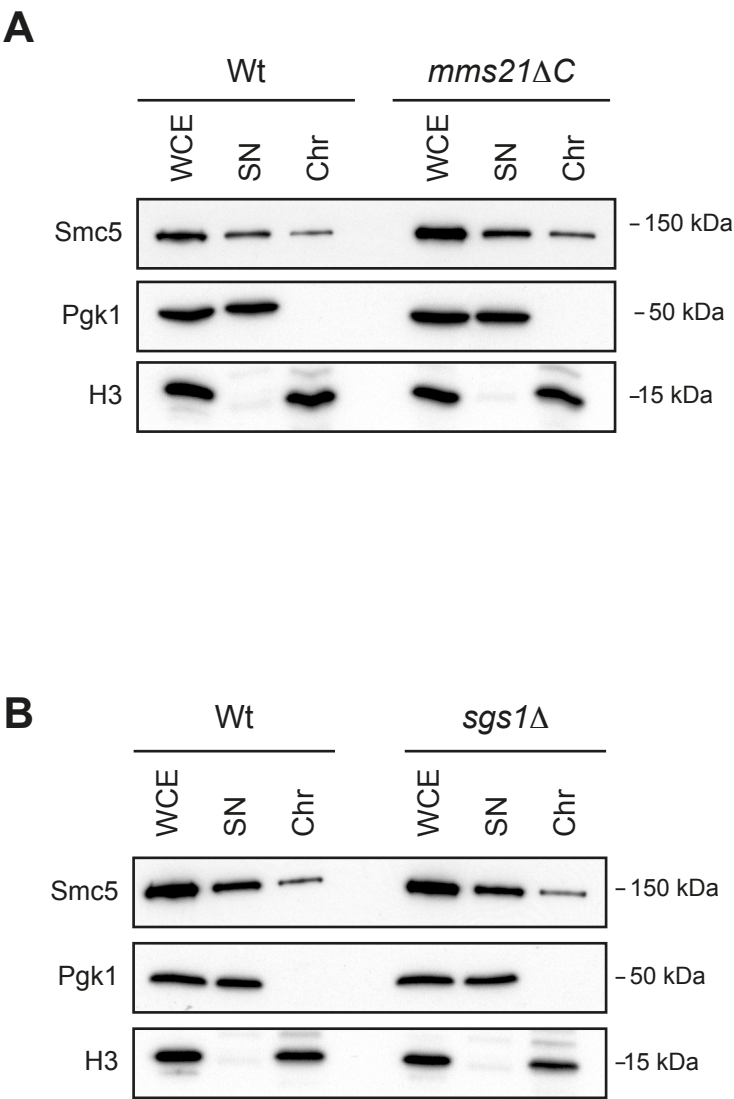

Supplement: Supplemental Material [file supp_30.11.1339_Supplemental_Figure_S8.pdf]

**Figure S9**

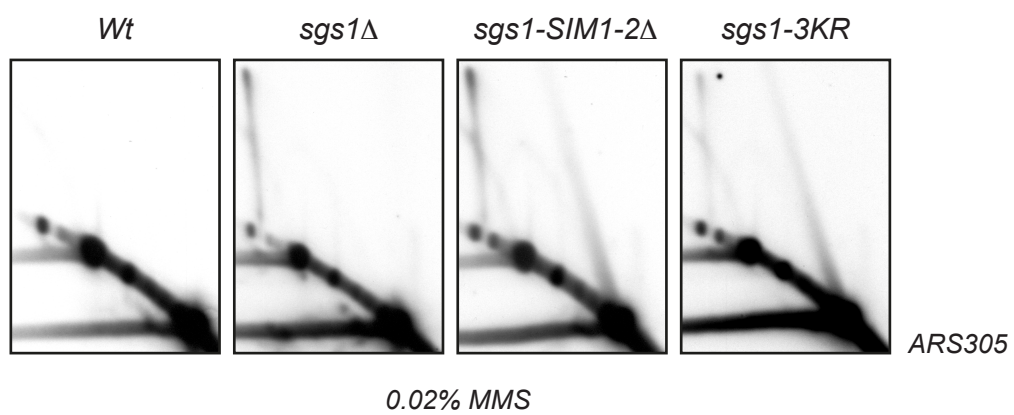

Supplement: Supplemental Material [file supp_30.11.1339_Supplemental_Figure_S9.pdf]
