# Supplementary material for: Sgs1's roles in DNA end resection, HJ dissolution, and crossover suppression require a two-step SUMO regulation dependent on Smc5/6
Source: Genes Dev. 2016 Jun 1;30(11):1339–56. doi: 10.1101/gad.278275.116 (PMC4911932; doi:10.1101/gad.278275.116)
Supplement: Supplemental Material [file supp_30.11.1339_Supplemental_Material.docx]

**SUPPLEMENTAL MATERIALS**

**FIGURE S1. Smc5, Sm6 and Nse4 are Mms21 substrates**

**A.** HF-SUMO pull-down from wild type, *mms21ΔC*, *siz1Δ*, *siz2Δ*, and *siz1-2Δ* cells expressing Smc5-9myc. **B.** HF-SUMO pull-down from wild type and *mms21ΔC* cells expressing Smc6-9myc. **C.** HF-SUMO pull-down from wild type and *mms21ΔC* cells expressing Nse4-9myc. Star points to unmodified form of proteins. Abbreviations: H-F-SUMO: His-Flag-SUMO; P.D: Pull-down.

**FIGURE S2. Sgs1 SUMO forms can be detected by HF-SUMO pull-down**

HF-SUMO pull-down from wild type cells expressing 6HisFlag-Smt3 and Sgs1-6HA. Cells were treated with 0.033% MMS for two hours before collecting them. Star points to unmodified form of Sgs1. Note the presence of high molecular bands only in the strain expressing the 6HisFlag-Smt3. Abbreviations: H-F-SUMO: His-Flag-SUMO; P.D: Pull-down.

**Figure S3. Sgs1 SUMOylation is Mms21-dependent**

**A.** HF-SUMO pull-down from wild type cells expressing Sgs1-6HA. Cells were treated with 0.033% or 0.3% MMS for two hours before collecting them. **B.** HF-SUMO pull-down from wild type and *mms21ΔC* cells expressing Sgs1-6HA. Cells were treated with 0.03% MMS for two hours before collecting them. Star points to unmodified form of Sgs1. Abbreviations: H-F-SUMO: His-Flag-SUMO; P.D: Pull-down.

**Figure S4**. **Sgs1 SUMOylation is partially Rad51-dependent and a single DSB triggers Sgs1 SUMOylation**

**A.** HF-SUMO pull-down from wild type and *rad51Δ *cells expressing Sgs1-6HA. Cells were treated with 0.0165% MMS for one hour before collecting them. **B.** HF-SUMO pull-down from wild type expressing Sgs1-6HA. Cells were grown overnight in YP raffinose. Then, the culture was split in two. One half was arrested in G1 with alpha factor and the other half in G2/M with nocodazole. Once arrested, galactose was added at a final concentration of 2% to induce HO expression and provoke a single DSB keeping them under arrest. Star points to unmodified form of Sgs1. Abbreviations: H-F-SUMO: His-Flag-SUMO; P.D: Pull-down.

**Figure S5**. **Sgs1 SUMOylation is impaired in *smc6-9* mutant cells**

HF-SUMO pull-down from wild type and *smc6-9* mutant cells expressing Sgs1-9myc. Cells were grown at 25ºC overnight, and they were shifted to 37ºC for 30 minutes to inactivate *smc6-9* allele. Then, cells were treated with 0.033% MMS for two hours before collecting them. Star points to unmodified form of Sgs1. Abbreviations: H-F-SUMO: His-Flag-SUMO; P.D: Pull-down.

**Figure S6**. **Smc5/6 is not involved in mediating interaction of STR subunits**

**A.** Analysis of Sgs1-Rmi1 interaction in wild type and *smc6-9* mutant cells. **B.** Analysis of Top3-Rmi1 interaction in wild type and *smc6-9* mutant cells. In a-b cells were grown overnight at 25ºC and shifted to 37ºC for 30 minutes to inactivate *smc6-9* mutant allele. Then, cells were treated with 0.033% MMS for two hours before collecting them. Star points to unspecific bands detected during the Co-Ip.

**Figure S7**. **Sgs1 recruitment to chromatin is not impaired in the *sgs1-3KR* mutant**

Chromatin fractionation assay from wild type cells expressing Sgs1-6HA wild type or sgs1-3KR. Cells were treated with 0.033% MMS for two hours before collecting them. Controls for a chromatin-bound protein (histone H3), cytoplasmic soluble (3-phosphoglycerate kinase; Pgk1) are shown. Quantification of the chromatin fractionation assays is shown. The abundance of Sgs1 on chromatin was normalized with histone H3, as an internal loading control in our blots. Mean values and standard deviations of two independent experiments are shown. Non-saturated exposures were used for gel quantifications using image J. Abbreviations: H-F-SUMO: His-Flag-SUMO; MMS, methyl methanesulfonate; WCE: whole cell extract; SN: supernatant; Chr: chromatin fraction.

**Figure S8. Smc5 recruitment to chromatin is not impaired in the *mms21ΔC* or in the *sgs1Δ* background**

**A.** Chromatin fractionation assay from cells expressing Smc5-9myc in a wild type or *mms21ΔC* background. **B.** Chromatin fractionation assay from cells expressing Smc5-9myc in a wild type or *sgs1Δ* background. Controls for a chromatin-bound protein (histone H3), cytoplasmic soluble (3-phosphoglycerate kinase; Pgk1) are shown. Abbreviations: H-F-SUMO: His-Flag-SUMO; MMS, methyl methanesulfonate; WCE: whole cell extract; SN: supernatant; Chr: chromatin fraction.

**Figure S9. Sgs1 SUMOylation promotes dissolution of HJs at damaged replication forks**

2D gel electrophoresis of wild type, *sgs1Δ*, *sgs1-SIM1-2Δ*, and *sgs1-3KR* mutant cells. Cells were arrested in G1 with α-factor. Once arrested, cells were released from G1 arrest into fresh media containing 0.02% MMS for 3 hours before samples were taken and processed for 2D gel. Note the accumulation of sister chromatid junctions in the mutant strains.

**Figure S10. Sgs1 SUMOylation is affected when fused to Ubc9 or Ulp1**

Western blot from cells expressing Sgs1-3HA, Sgs1-Ubc9-3HA, or Sgs1-Ulp1-3HA.

Cells were grown overnight in YP raffinose. Then, galactose was added to a final concentration of 2% and the culture was split in two. One half was treated with 0.033% MMS for two hours before collecting them.

**Table S1.** Relevant genotype of yeast strains used in this study

| AS499 | *MATa bar1Δ leu2-3,112 ura3-52 his3Δ200 trp1-Δ63 ade2-1 lys2-801 pep4* |
| --- | --- |
| BY4733 | *MATa his3Δ200 leu2Δ0 met15Δ0 trpΔ63 ura3Δ0* |
| BY4741 | *MATa his3Δ1 leu2Δ0 met15Δ0 ura3Δ0* |
| W3O3 | *MATa ho ade2-1 trp1-1 can1-100 leu2-3,112 his3-11,15 ura3-1* |
| YTR28 | BY4733 + *SMC6-9myc:TRP* |
| YTR83 | AS499 + *NSE4-9myc:TRP* |
| YTR557 | BY4741 + *6His-Flag-SMT3:kanMX6* |
| YTR570  YTR793 | BY4741 + *6His-Flag-SMT3:kanMX6 mms21ΔC:hphMx4*  BY4741 + *6His-Flag-SMT3:kanMX6 mms21ΔC:hphMx4 SMC5-9myc:HIS3* |
| YTR794 | BY4741 + *6His-Flag-SMT3:kanMX6 SMC5-9myc:HIS3* |
| YMB811 | BY4741 + *6His-Flag-SMT3:kanMX6 siz1::hphMx4 SMC5-9myc:HIS3* |
| YMB813 | BY4741 + *6His-Flag-SMT3:kanMX6 siz2::natNT SMC5-9myc:HIS3* |
| YMB823 | BY4741 + *6His-Flag-SMT3:kanMX6 siz1::natNT siz2::hphMx4*  *SMC5-9myc:HIS3* |
| YTR844 | BY4733 + *6His-Flag-SMT3:kanMX6 SMC6-9myc:TRP* |
| YTR846 | AS499 + *6His-Flag-SMT3:kanMX6 NSE1-9myc:TRP* |
| YTR848 | AS499 + *6His-Flag-SMT3:kanMX6 NSE6-9myc:TRP* |
| YTR850 | AS499 + *6His-Flag-SMT3:kanMX6 NSE5-9myc:TRP* |
| YTR852 | AS499 + *6His-Flag-SMT3:kanMX6 MMS21-9myc:TRP* |
| YTR854 | AS499 + *6His-Flag-SMT3:kanMX6 NSE3-9myc:TRP* |
| YTR856 | AS499 + *6His-Flag-SMT3:kanMX6 NSE4-9myc:TRP* |
| YMB906 | BY4733 + *6His-Flag-SMT3:kanMX6 mms21ΔC:hphMx4 SMC6-9myc:HIS3* |
| YMB1110 | BY4741 + *6His-Flag-SMT3:kanMX6 mms21ΔC:hphMx4 NSE4-9myc:HIS3* |
| CCG4620  CCG7813  CCG9895 | BY4741 + *6His-Flag-SMT3:kanMX6*  BY4741  *hoΔ hml::ADE1 MATa-inc hmr::ADE1 ade1 leu2-3,112 lys5 trp1::hisG ura3-52 ade3::GAL::HO arg5,6::MATa::HPH* |
| CCG9896 | *hoΔ hml::ADE1 MATa-inc hmr::ADE1 ade1 leu2-3,112 lys5 trp1::hisG ura3-52 ade3::GAL::HO arg5,6::MATa::HPH sgs1::kanMX6* |
| CCG9928  CCG9929 | *MATa ade1-100 leu2,3-112 lys5 ura3-52 trp1::hisG hoΔ hml::ADE1 hmr::ADE1 ade3::GAL-HO exo1::kanMX6 sgs1::hphMx4*  *MATa ade1-100 leu2,3-112 lys5 ura3-52 trp1::hisG hoΔ hml::ADE1 hmr::ADE1 ade3::GAL-HO exo1::kanMX6* |
| CCG10418 | AS499 *+ sgs1::hphMx4* |
| CCG10437 | AS499 *+ sgs1::hphMx4 pRS402-GALp-SGS1:3HA* |
| CCG10439 | AS499 *+ sgs1::hphMx4 pRS402-GALp-SGS1-cULP1(F474A-C580S):3HA* |
| CCG10441 | AS499 *+ sgs1::hphMx4 pRS402-GALp-SGS1-cULP1:3HA* |
| CCG10443 | AS499 *+ sgs1::hphMx4 pRS402-GALp-SGS1-UBC9(C93S):3HA* |
| CCG10445 | AS499 *+ sgs1::hphMx4 pRS402-GALp-SGS1-UBC9:3HA* |
| CCG10659 | BY4741 + *6His-Flag-SMT3:kanMX6 smc6-9:natNT SGS1-9myc:hphMx4* |
| CCG10669 | BY4741 + *6His-Flag-SMT3:kanMX6 SGS1-9myc:hphMx4* |
| CCG10704  CCG10734 | BY4741 + *6His-Flag-SMT3:kanMX6 mms21ΔC:hphMx4 SMC5-9myc:HIS3 SGS1-6HA::natNT*  AS499 + *SGS1-6HA:hphMx4* |
| CCG10736 | *hoΔ hml::ADE1 MATa-inc hmr::ADE1 ade1 leu2-3,112 lys5 trp1::hisG ura3-52 ade3::GAL::HO arg5,6::MATa::HPH smc6-9:natNT* |
| CCG10787 | AS499 + *SGS1-6HA:hphMx4 TOP3-9myc:His3* |
| CCG10788 | AS499 + *SGS1-6HA:hphMx4 RMI1-9myc:His3* |
| CCG10792 | AS499 + *TOP3-6HA:hphMx4 RMI1-9myc:His3* |
| CCG10799 | BY4741 + *6His-Flag-SMT3:kanMX6 siz1::natNT siz2::hphMx4*  *SGS1-6HA:HIS3* |
| CCG10803 | BY4733 + *SMC5-9myc:TRP TOP3-6HA:hphMx4 mms21ΔC:natNT* |
| CCG10810 | BY4741 + *6His-Flag-SMT3:kanMX6 TOP3-6HA:hphMx4* |
| CCG10868 | BY4733 + *SMC5-9myc:TRP SGS1-6HA:HIS3* |
| CCG10870 | BY4733 + *SMC5-9myc:TRP TOP3-6HA:hphMx4 sgs1::natNT* |
| CCG10882 | AS499 + *6His-Flag-SMT3:kanMX6 RMI1-6HA:hphMx4* |
| CCG10883 | BY4733 + *SMC5-9myc:TRP TOP3-6HA:hphMx4* |
| CCG10884 | BY4733 + *SMC5-9myc:TRP SGS1-6HA:HIS3 smc6-9:natNT* |
| CCG10885 | BY4741 + *6His-Flag-SMT3:kanMX6 TOP3-6HA:hphMx4 mms21ΔC:natNT* |
| CCG10889 | AS499 + *SGS1-6HA:hphMx4 TOP3-9myc:HIS3 mms21ΔC:natNT* |
| CCG10898 | AS499 + *SGS1-6HA:hphMx4 RMI1-9myc:HIS3 smc6-9:natNT* |
| CCG10919 | AS499 + *RMI1-9myc:HIS3 TOP3-6HA:hphMx4 smc6-9:natNT* |
| CCG10922 | AS499 + *RMI1-9myc:HIS3 TOP3-6HA:hphMx4 mms21ΔC:natNT* |
| CCG10957  CCG10973 | AS499 + *6His-Flag-SMT3:kanMX6 sgs1::hphMx4*  AS499 + *TOP3-9myc:HIS3 pRS402-GALp-SGS1:3HA* |
| CCG10975  CCG10979  CCG10982  CCG11062 | AS499 + *6His-Flag-SMT3:kanMX6 sgs1::hphMx4 pRS402-GALp-SGS1:3HA*  AS499 + *6His-Flag-SMT3:kanMX6 sgs1::hphMx4 pRS402-GALp-SGS1-cULP1:3HA*  AS499 + *6His-Flag-SMT3:kanMX6 sgs1::hphMx4 pRS402-GALp-SGS1-UBC9:3HA*  BY4741 + *6His-Flag-SMT3:kanMX6 SGS1-6HA:HIS3* |
| CCG11253 | *W3O3* + *6His-Flag-SMT3:kanMX6 SGS1-6HA:hphMx4* |
| CCG11254 | W3O3 + *rad53::HIS3 sml1-1 6His-Flag-SMT3:kanMX6 SGS1-6HA:hphMx4* |
| CCG11409 | AS499 + *6His-Flag-SMT3:kanMX6 SGS1-6HA:HIS3* |
| CCG11791 | AS499 + *6His-Flag-SMT3:kanMX6 sgs1-K621R-6HA:hphMx4* |
| CCG11830 | AS499 + *ubc9-1:TRP 6His-Flag-SMT3:kanMX6 SGS1-6HA:hphMx4* |
| CCG11835 | BY4741 + *6His-Flag-SMT3:kanMX6 SGS1-6HA:HIS3 rad51::hphMx4* |
| CCG12042 | AS499 + *SMC5-9myc:natNT pRS402-GALp-SGS1(SIM1Δ):3HA* |
| CCG12045 | AS499 + *SMC5-9myc:natNT pRS402-GALp-SGS1(SIM1-2Δ):3HA* |
| CCG12118 | AS499 + *SMC5-9myc:natNT pRS402-GALp-SGS1:3HA* |
| CCG12155 | AS499 + *6His-Flag-SMT3:kanMX6 sgs1-3KR-6HA:hphMx4* |
| CCG12415 | BY4741 + *6His-Flag-SMT3:kanMX6 SGS1-6HA:HIS3 mms21ΔC:hphMx4* |
| CCG12483 | AS499 + *TOP3-9myc:HIS3 pRS402-GALp-SGS1(SIM1Δ):3HA* |
| CCG12484 | AS499 + *TOP3-9myc:HIS3 pRS402-GALp-SGS1(3KR):3HA* |
| CCG12486 | AS499 + *SMC5-9myc:natNT pRS402-GALp-SGS1(3KR):3HA* |
| CCG13154 | AS499 + *6His-Flag-SMT3:kanMX6 sgs1-SIM1-2Δ-6HA:hphMx4* |
| CCG13161 | AS499 + *6His-Flag-SMT3:kanMX6 sgs1-SIM1Δ-6HA:hphMx4* |
| CCG13196 | *MATa ade1-100 leu2,3-112 lys5 ura3-52 trp1::hisG hoΔ hml::ADE1 hmr::ADE1 ade3::GAL-HO exo1::kanMX6 sgs1-SIM1-2Δ-6HA:hphMx4* |
| CCG13228 | *MATa ade1-100 leu2,3-112 lys5 ura3-52 trp1::hisG hoΔ hml::ADE1 hmr::ADE1 ade3::GAL-HO exo1::kanMX6 sgs1-K621R-6HA:hphMx4* |
| CCG13229 | AS499 + *mms4::natNT* |
| CCG13233 | AS499 + *6His-Flag-SMT3:kanMX6 sgs1-3KR-6HA:hphMx4 mms4::natNT* |
| CCG13327 | AS499 + *6His-Flag-SMT3:kanMX6 sgs1-SIM1-2Δ-6HA:hphMx4 mms4::natNT* |
| CCG13450 | *hoΔ hml::ADE1 MATa-inc hmr::ADE1 ade1 leu2-3,112 lys5 trp1::hisG ura3-52 ade3::GAL::HO arg5,6::MATa::HPH sgs1::kanMX6 pRS406-SGS1p-SGS1:6HA* |
| CCG13453 | *hoΔ hml::ADE1 MATa-inc hmr::ADE1 ade1 leu2-3,112 lys5 trp1::hisG ura3-52 ade3::GAL::HO arg5,6::MATa::HPH sgs1::kanMX6 pRS406-SGS1p-SGS1(3KR):6HA* |

CCG13627 *hoΔ hml::ADE1 MATa-inc hmr::ADE1 ade1 leu2-3,112 lys5 trp1::hisG ura3-52 ade3::GAL::HO arg5,6::MATa::HPH sgs1-SIM1-2Δ-6HA:hphMx4*

CCG13662 MATa ade2-1 ura3-1 his3-11, 15 trp1-1 can1-100 ade3::pGAL::HO Hocs::KanMX near PES4 (*ARS604*) hmlΔ::OropRS hmrΔ::ampr matHOcsD::pBR bar1Δ::TRP1 *6His-Flag-SMT3:kanMX6 SGS1-6HA::natNT*

**Yeast strains used in this study per figure:**

**Fig. 1A:** YTR557, YTR570, BY4741. **Fig. 1B:** YTR557, YTR794, YTR844, YTR846, YTR848, YTR850, YTR852, YTR854, YTR856. **Fig. 1C:** 4620, 7813, 10734, 11409. **Fig. 1D:** 10734, 11409. **Fig. 1E:** 11409, 11830. **Fig. 1F:** 11062, 10799. **Fig.1G:** 11062, 12415. **Fig. 2A:** 10882. **Fig. 2B:** 4620, 7813, 10810, 10883. **Fig. 2C:** 10810, 10885. **Fig. 2D:** 11409. **Fig. 2E:** 11062. **Fig. 2F:** 11253, 11254. **Fig. 2G:** 11062, 11835. **Fig. 2H:** 11062. **Fig. 2I:** 11062. **Fig. 3A:** 11062, 10868. **Fig. 3B:** 11062, 10868. **Fig. 3C:** 10810, 10883. **Fig. 3D:** 11062, 10868, 10884. **Fig. 3E:** 10803, 10810, 10883. **Fig. 3F:** 10810, 10883, 10870. **Fig. 3G:** 10734, 10787, 10889. **Fig. 3H:** 10792, 10810, 10922. **Fig. 4B:** 11409, 13154, 13161. **Fig. 4C:** 10437, 12042, 12045, 12118. **Fig. 4D:** 10437, 10973, 12483. **Fig. 4E:** AS499, 12072, 12118. **Fig. 5A:** 11409, 11791. **Fig. 5B:** 11409, 11791, 12155. **Fig. 5C:** 10437, 12118, 12486. **Fig. 5D:** 10437, 10973, 12484. **Fig. 5E:** AS499, 12118, 12486. **Fig. 5F:** 11062, 12415. **Fig. 5G:** 10973, 12484. **Fig. 5H:** 12118, 12045. **Fig. 6A:** 11409, 13154. **Fig. 6B:** 10418, 11409, 12155. **Fig. 6C:** 11409, 10418, 12155, 13229, 13233. **Fig. 6D:** 11409, 10418, 13154, 13229, 13227. **Fig. 6E:** 10418, 10437, 10445, 10443, 10441, 10439. **Fig. 7B:** 9895, 9896, 10736. **Fig. 8B:** 13450, 13453. **Fig. 9A:** 9928, 9929. **Fig.9B:** 9929, 13196, 13228. **Fig.S1A:** YTR557, YTR794, YTR793, YMB811, YMB813, YMB823. **Fig. S1B:** YTR557, YTR28, YTR844, YMB906. **Fig. S1C:** YTR557, YTR83, YTR856, YTR1110. **Fig. S2:** 10734, 11409. **Fig. S3A:** 11062. **Fig. S3B:** 11062, 12415. **Fig. S4A:** 11062, 11835. **Fig. S4B:** 13662. **Fig. S5:** 10659, 10669. **Fig. S6A:** 10734, 10788, 10898. **Fig. S6B:** 10810, 10792, 10919. **Fig. S7:** 11409, 12155. **Fig. S8A:** 10868, 10704. **Fig. S8B:** 10883, 10870. **Fig. S9:** 11409, 10418, 12155, 13154. **Fig. S10:** 10957, 10975, 10982, 10979. **Fig. S11:** 9895, 13627.

**Table S2.** Plasmids used in this study

| 262 | *pRS402-GALp-SGS1:3HA* |
| --- | --- |
| 263 | *pRS402-GALp-SGS1-cULP1(F474A-C580S):3HA* |
| 264 | *pRS402-GALp-SGS1-cULP1:3HA* |
| 266 | *pRS402-GALp-SGS1-UBC9(C93S):3HA* |
| 267 | *pRS402-GALp-SGS1-UBC9:3HA* |
| 268 | *pRS402-GALp-SGS1(3KR):3HA* |
| 273 | *pRS402-GALp-SGS1(SIM1Δ):3HA* |
| 276 | *pRS402-GALp-SGS1(SIM1-2Δ):3HA* |
| 515 | *pRS406-SGS1p-SGS1:6HA* |
| 519 | *pRS406-SGS1p-SGS1(3KR):6HA* |
